# Cancer‐Targeting Peptides Functionalized With Polyarginine Enables GRP78‐Dependent Cell Uptake and siRNA Delivery Within the DU145 Prostate Cancer Cells

**DOI:** 10.1002/psc.70007

**Published:** 2025-02-18

**Authors:** George Hilan, Grace Daniel, Filiz Collak, David Sabatino, William G. Willmore

**Affiliations:** ^1^ Department of Biology Carleton University Ottawa ON Canada; ^2^ Department of Chemistry Carleton University Ottawa ON Canada; ^3^ Institute of Biochemistry Carleton University Ottawa ON Canada

**Keywords:** cancer‐targeting peptides, cell‐penetrating peptides, DU145 cells, GRP78, polyarginine, siRNA

## Abstract

This study investigated a peptide‐based GRP78‐targeting strategy for short‐interfering (si) RNA delivery in cancer cells. Synthetic fluorescein‐labeled amphiphilic peptides composed of the hydrophobic cell surface (cs) GRP78‐targeting and hydrophilic, polycationic arginine‐rich cell penetrating peptides demonstrated GRP78‐dependent cell uptake in the DU145 prostate cancer cells, and to a lesser extent in the non‐cancerous human lung fibroblast WI‐38 cell line. Mechanistic studies revealed energy‐dependent GRP78 receptor‐mediated endocytosis of the GRP78‐targeting peptide with polyarginine (W1‐R9). The cytosolic accumulation of this peptide underscored its potential utility in siRNA delivery. Peptide:siRNA complexes formed stably condensed nanoparticles, with calcium functioning as an ionic stabilizer and additive promoting endosomal siRNA escape for RNA interference (RNAi) activity. Preliminary peptide‐based siRNA transfections in the DU145 cells demonstrated that GRP78 knockdown led to an interplay in between pro‐survival and cell death outcomes under ER stress induction. Thus, the GRP78‐targeting polyarginine peptides enables efficient cell uptake for specific siRNA delivery in the DU145 cells. This class of bio‐active synthetic peptides is important for the investigation of cancer biology, leading to the innovation of cancer‐targeted gene delivery and therapy approaches.

## Graphical abstract

The integration of a polyarginine cell penetrating peptide with a GRP78‐targeting peptide provides selective and effective peptide‐based siRNA transfection directly within the GRP78 overexpressing prostate cancer (DU145) cells.

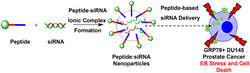

## 1 Introduction

The Glucose Regulated Protein of 78 kilodaltons (GRP78) is also known as the immunoglobulin heavy chain binding protein (BiP/HSPA5), and is a member of the heat shock protein 70 (HSP70) family of stress‐inducible chaperones [1]. As chaperone protein, GRP78 has a subcellular localization in the lumen of the endoplasmic reticulum (ER) where it plays critical roles in the regulation of protein quality control, by folding proteins that follow the excretory pathway and exporting misfolded proteins into the cytosol for ubiquitination and proteasomal degradation [2]. Thus, GRP78 acts as the “master” regulator of the unfolded protein response (UPR; also known as the ER stress response), and a main sensor of the accumulation of unfolded proteins within the ER lumen and activating ER‐associated degradation (ERAD) by the proteasome [3]. In this manner, GRP78 regulates anti‐apoptotic (pro‐survival) and apoptotic cellular functions. Furthermore, GRP78 also balances calcium (Ca^2+^) flux from the ER to the mitochondria to maintain cellular bioenergetics for metabolic homeostasis and cell survival [4]. In this mechanism, GPR78 adapts the UPR to ensure a pro‐survival outcome and suppression of stress‐induced apoptosis by salvaging protein quality control and bioenergetics during ER stress [4, 5]. Alternatively, under prolonged ER stress, GRP78 activates the UPR‐dependent cell death pathways [6, 7, 8, 9, 10]. Thus, GRP78 plays a critical role in the regulation of cell survival and apoptosis during ER stress induction.

GRP78 is found to be highly expressed in diseases associated with a broad variety of cellular stresses, such as hyperproliferation, ROS, hypoxia, DNA damage and nutrient (glucose) deprivation [11]. For example, GRP78 overexpression confers pro‐survival/oncogenic signaling [12], associated with rapid cell metabolism, proliferation, adhesion, invasion and spread, that function as characteristic hallmarks of cancer [13]. Various malignant tissues, including lung, breast, prostate, and liver cancers possess high GRP78 levels in the tumor microenvironment [14]. The overexpression of GRP78 also promotes translocation to the cell surface, where cell surface (cs) GRP78 acts as a multifunctional receptor, that regulates cell proliferation, survival and anti‐apoptotic pathways contributing to tumor progression [15]. Furthermore, csGRP78 occurs primarily on malignant, tumor cell types, but is absent or minimally expressed on the surface of non‐malignant cells and tissues, making it a therapeutic biological marker (biomarker) for the innovation of precision oncology (anti‐cancer) strategies [16, 17].

The discovery of csGRP78 binding peptide ligands as cancer‐targeting peptides (CTPs) has enabled direct therapeutic intervention within GRP78 overexpressing tumors [18]. For example, the selection of GPR78 specific peptides using phage display uncovered two nonpolar peptides (W1, WIFPWIQL and D, WDLAWMFRLPVG) that maintained the most favorable binding activity to csGRP78 presenting human prostate cancer (DU145) cells [19]. However, nonpolar peptides have limited cell uptake efficiency, thereby restricting their drug delivery applications. Extension of the hydrophobic W1 peptide with a polycationic programmed cell death‐inducing sequence, D‐(KLAKLAK)_2_, enabled GRP78‐dependent cell uptake and apoptosis within the DU145 cells, leading to tumor growth inhibition in a xenograft prostate cancer model, underscoring its potential therapeutic utility [19]. Thus, the hydrophobic GRP78‐targeting peptides can be functionalized with polar, polycationic cell‐penetrating peptides that enhance cell uptake for selective and efficient drug delivery in csGRP78‐presenting tumors.

Cell penetrating peptides (CPP) are typically short (< 30 amino acids) sequences capable of translocation across cell membranes, making them potentially useful carriers in drug delivery applications [20, 21]. Cationic CPPs such as the human immunodeficiency virus 1 (HIV‐1) TAT (RKKRRQRRR) or polyarginine (e.g., R9) sequences, can also contain non‐polar (Trp, His, or Ala) residues, that enable favorable electrostatic and nonpolar interactions with the amphiphilic lipid bilayer, that ultimately disrupts the plasma membrane for cell translocation [22]. The mechanism of cell uptake for CPPs relies on sequence composition, charge (ionic) and secondary structures that may also favor self‐assembly of stably condensed (nano)particles for cell delivery [23]. Previous work revealed the GRP78‐dependent cell uptake of disulfide‐linked GRP78‐targeting polyarginine peptides, (CTVALPGGYVRVC‐Rn) in the HepG2 hepatoblastoma cell line, in which cell binding and translocation activity was contingent on the length of the polyarginine sequence (Rn = 3–12) [24]. Improved cell binding and uptake efficacy was detected with lengthier (R9‐R12) vs. shorter (R3–R6) polyarginine CPP sequence, underscoring the importance of peptide composition on biological activity. Alternatively, synthetic peptide modifications, including cyclization and acylation, can impart significant improvements in peptide cell uptake efficiency, even with shorter length (R5‐R6) polyarginines [25, 26]. Thus, the arginine‐rich CPPs can undergo cell uptake with various efficacy, that is contingent on the amphiphilic, polycationic sequence and structure compositions.

CPPs with hydrophobic and polycationic (Lys, Arg, Trp and His) residues, have been reported for gene delivery in vitro and in vivo [27]. For example, arginine‐rich CPPs have served as efficient short‐interfering (si)RNA carrier systems due to their cationic nature under physiological conditions. Polyarginine CPPs (e.g., R9) favor electrostatic interactions with the negative charged phosphodiester backbone of nucleic acids, enabling condensation into ionic (nano)particles for membrane recognition, translocation and endosomal release of siRNA into the cytosol of cells for RNA interference (RNAi) activity [28, 29]. In human gastric carcinoma cells, polyarginine R9 with enhanced green fluorescent protein (eGFP) siRNA demonstrated effective eGFP silencing, by a decrease in green fluorescence in treated cells compared to the untreated controls [29]. Effective in vivo RNAi activity was also achieved in a mouse model bearing subcutaneous human ovarian SKOV‐3 cancer cells. Reduction in tumor growth was detected after intratumoral administration of HER‐2‐targeting siRNA/peptide complexes [30]. Thus, arginine‐rich peptides are a promising class of CPPs for siRNA delivery and gene silencing therapies. However, these peptides are widely dispersed in all cell types by various cell uptake mechanisms, thereby restricting their capacity to deliver therapeutic cargo selectively within in vitro and in vivo tumor systems [31].

**FIGURE 1:**
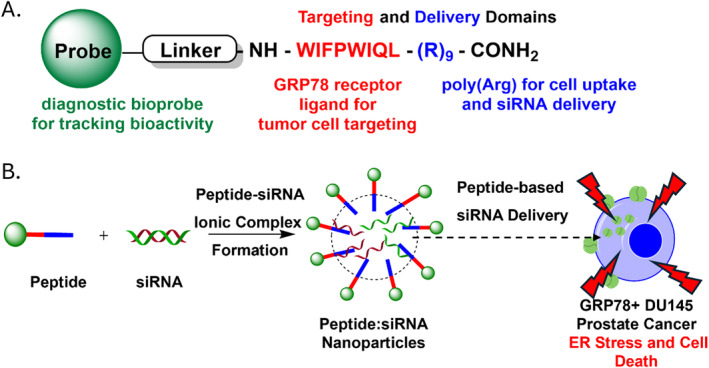
GRP78‐targeting approach. A. Design of GRP78‐targeting polyarginine peptides for siRNA delivery. B. Formulation strategy of peptide:siRNA nanoparticles for GRP78 silencing activity in the DU145 prostate cancer cells.

This work is built upon the combination of the polyarginine (R9) CPP with the GRP78‐targeting (W1) peptide, for improving cell uptake efficiency and specificity directly within the GRP78 overexpressing (DU145) prostate cancer cells. The amphiphilic polyarginine‐derived GRP78‐targeting peptide sequence (W1‐R9) is anticipated to undergo an energy‐dependent cell uptake mechanism via receptor‐mediated endocytosis. This GRP78‐dependent mechanism of cell uptake is correlated with other GRP78‐targeting peptides [19, 32, 33, 34]. Improved cell uptake efficiency by the incorporation of the polyarginine (R9) CPP is anticipated to favor peptide‐GRP78 receptor membrane fusion, formation of endosomes for peptide translocation and intracellular localization based on the cell entry mechanisms of arginine‐rich peptides [22, 23, 24]. Considering polycationic polyarginine CPPs also enable stable and reversible ionic interactions with gene (e.g., siRNA) therapeutics, the GRP78‐targeting polyarginine peptide (W1‐R9) may also be applicable to siRNA delivery and silencing activity in the GRP78‐overexpressing DU145 prostate cancer cells (Figure 1). Interestingly, GRP78 knockdown promoted ER stress induction that also revealed a correlation in between pro‐survival and programmed cell death (apoptosis) responses in the human DU145 prostate cancer cells, underscoring the potential anti‐cancer utility of this GRP78‐targeting approach.

## 2 Materials and Methods

### 2.1 Materials

The human, male prostate cancer (adenocarcinoma) cell line (DU145 cells; ATCC HTB‐81) was obtained as a gift from Dr. Bruce McKay at Carleton University. Fetal human lung fibroblasts (WI‐38 cells) were obtained from the American Type Culture Collection (ATCC, CCL‐75). The cell culturing conditions used MultiCell's Essential Modified Eagle's Medium (EMEM, ThermoFisher, CAS: 320‐005‐CL) supplemented with 10% heat inactivated fetal bovine serum (FBS, ThermoFisher). The 3‐(4,5‐dimethylthiazol‐2‐yl)‐2,5‐diphenyl tetrazolium bromide (MTT, BioShop, CAS: 298–93‐1) reagent was used for cell viability. Glass coverslips (ThermoFisher, Cat #: 12541016CA – 22 × 22 mm) were sterilized with ethyl alcohol and coated with 1× poly‐L‐lysine (Millipore Sigma, CAS: 25988‐63‐0) for confocal microscopy. For cell localization studies, MitoTracker Deep Red (Invitrogen – M22426), Alexa Fluor 647 Phalloidin (Invitrogen – A22287) and DAPI histology mounting media (Millipore Sigma F6057, Fluoroshield™ with DAPI), were respectively used as mitochondrial, actin and nuclear stains. The endocytosis inhibitors, chlorpromazine (EMD Millipore, CAS: 69‐09‐0) and methyl‐β‐cyclodextrin (EMD Millipore, CAS: 128446‐36‐6), were used directly as purchased. The primary antibodies, mouse monoclonal GRP78 antibody (ThermoFisher, 1D6F7, Cat #: 66574‐1‐IG), mouse anti‐CHOP monoclonal antibody (ThermoFisher, Cat#: MA1‐250), unspliced rabbit‐anti XBP1 polyclonal antibody (Novus Bio., NBP1‐77681), mouse beta‐actin monoclonal antibody (Sigma A5316) and rabbit anti‐GAPDH monoclonal antibody (ABclonal, A19056), as well as secondary antibodies, anti‐rabbit HRP conjugated antibody (Santa Cruz Biotech., ac‐2357) and anti‐mouse HRP conjugated antibody (Dako, P0447) were used as received. The BCA reagent (ThermoFisher, 23223) and copper(II) sulfate reagent B (ThermoFisher, C2284‐25 mL) were used for protein quantitation. Western blot was performed in Laemmli sample buffer (BioRad, Cat#: 1610737) with β‐mercaptoethanol (Millipore Sigma), with immunoblot transfer on a PVDF membrane (BioRad, Cat#: 1620177) and blocking with 5% dry milk in 1× Tris‐buffered saline‐Tween 20 (TBST, ThermoFisher). A Clarity Western ECL substrate Chemileuminences (BioRad, Cat#: 170‐5060) was used to visualize the membranes. A Bio‐Rad Aurum Total RNA mini–Isolation Kit (BioRad, Cat#: 7326820) was used for RNA isolation. The isolated RNA was converted to cDNA using the Bio‐Rad iScript cDNA Synthesis Kit (Cat # 1708890). The primers for GAPDH (housekeeping gene), GRP78, CHOP, caspase 3, and spliced XBP1 and ATF4 were ordered from IDT, with the corresponding sequences shown in Table S1, ESI. For qPCR, a Bio‐Rad SsoAdvanced Universal SYBR Green Supermix (Cat #: 1725271) was used for each reaction. The reactions were amplified using a BioRad CFX connect Real‐Time system qPCR machine using the protocol provided in the Bio‐Rad manual for the SYBR Green kit.

### 2.2 Molecular Modeling and Docking

The crystal structure of the GRP78 substrate binding domain (Asp 418 – Gly 652) was available from the Protein Data Bank (PDB ID # 5E85). A molecular docking study was performed to visualize the binding model of the peptides in the binding pocket of GRP78. An interface between the molecular graphics system PyMOL and the molecular docking suite AutoDockVina was used to demonstrate the combination of docking and visualization models. The peptide was enclosed in a box with number of grid points in x, y and z directions, 50 × 50 × 50 and a grid spacing of 0.375 Å. The center of the grid was set to −0.352, 59.611, and 53.093. For the docking studies, the lowest energy docked conformation, according to the AutoDock scoring function was selected as the most stable peptide binding model.

### 2.3 TEM

Peptide:siRNA samples were prepared at a 5:1 N:P ratio in milliQ H_2_O according to an established literature protocol [28]. Samples were also incubated with 50 mM CaCl_2_ (5 μL) for 30 min at room temperature. An additional aliquot (7.5 μL) of 1% uranyl acetate in milliQ H_2_O was added to the samples to ensure equivalent volumes. Aliquots (10 μL) of individual samples were then deposited onto a 300‐mesh formvar copper grid coated with carbon (Electron Microscopy Sciences Inc., Hatfield, PA). It was allowed to evaporate for 1 h and TEM images were obtained using the FEI Tecnai G2 F20 transmission electron microscope at an accelerating voltage of 120 kV and the Gatan ORIUS TEM CCD camera was used to capture images across the grids.

### 2.4 Cell Culture

DU145 and WI‐38 cells were grown in Essential Modified Eagle's Medium (EMEM; Gibco) supplemented with 10% heat inactivated fetal bovine serum (10% FBS) and incubated at 37°C, in a ThermoForma triple gas (N_2_‐O_2_‐CO_2_) incubator set at 21% O_2_ and 5% CO_2_, with 95% relative humidity. All cell work was conducted in a biosafety cabinet (ThermoScientific – 1300 Series A2 Hood).

### 2.5 Cell Viability

The 3‐(4,5‐dimethylthiazol‐2‐yl)‐2,5‐diphenyl tetrazolium bromide (MTT, BioShop, CAS: 298‐93‐1) cell viability reagent was used in 1× phosphate buffered saline (PBS) at 5 mg/mL stock volume and filtered through a 0.22 μm filter. For the MTT assay, cells were seeded in 96‐well plates at 20,000 cells per well and incubated at 37°C for 24 h. After seeding, cells were treated, then washed with 1× PBS twice before adding 100 μL of serum free EMEM with 10% v/v of the filtered MTT solution (10 μL). The cells were incubated with MTT for 4 h, at 37°C. Following incubation, the MTT containing media was carefully removed, to retain the purple formazan crystals, and of dimethyl sulfoxide (DMSO, 100 μL) was added to dissolve the crystals. The plates were incubated at 37°C for 1 h, then imaged on a (CYTATION|5 Imaging Reader) plate reader at 570 nm. The results were exported and processed on an Excel spreadsheet for data analysis.

### 2.6 Flow Cytometry

The DU145 cells were detached from a T‐75 cm flask using 1× trypsin (1 mL, 3 min), followed by addition of EMEM (4 mL) supplemented with 10% FBS. The cells were collected in a 15 mL canonical tube, and spun down in a (MIKRO 220 Hettich) centrifuge at 1500 rpm for 5 min. The cell pellet was washed twice with serum free EMEM to remove FBS. Finally, the cells were suspended in serum free EMEM (4 mL), and analyzed for cell counting, sorting and viability by Trypan Blue (Millipore Sigma; T8154) staining and visualized under a Zeiss Axiovert 200 brightfield microscope at 10× magnification. The DU145 cells (~1 × 10^6^) were added to microcentrifuge tubes, followed by addition of FITC‐labelled peptides (10 μM in milli‐Q H_2_O) to individual tubes for flow cytometry analysis on the BD Accuri C6 flow cytometer, measuring peptide direct binding on the DU145 cells. For competitive binding/inhibition studies, the primary monoclonal GRP78 antibody (ThermoFisher – 1D6F7) was added (2 μg/mL) to the DU145 cells to bind to csGRP78 at 37°C for 1 h in a rotating mixer. After which, the peptides were added (10 μM in milli‐Q H_2_O) and allowed to incubate at 37°C for 30 min in a rotating mixer. The cells were then washed using 1X chilled PBS, then centrifuged at 1300 rpm and resuspended in cold 1X PBS for flow cytometry analysis with FITC channel detection set at 451 nm.

### 2.7 Confocal Fluorescence Microscopy

Glass coverslips (ThermoFisher, 22 × 22 mm) were sterilized with ethyl alcohol and coated with 1× poly‐L‐lysine (Millipore Sigma) and incubated at 37°C for 15 min. The poly‐l‐lysine was then collected, and the slides were washed three times with 1× PBS, then dried for 1 h under UV light. DU145 cells were then counted and seeded at a density of 3.5 × 10^5^ cells per 35 cm plate for 24 h. For cell localization studies, the DU145 or WI‐38 cells were treated with 50 nM of MitoTracker Deep Red (Invitrogen – M22426 – λ_ex_ = 579 nm, λ_em_ = 599 nm) for 30 min, then washed with 1× PBS twice to remove excess Mito Tracker. This was followed by an incubation with the desired peptide (10 μM in milli‐Q H_2_O) for 30 min. Cells were washed twice with 1× PBS, and then fixed with 4% paraformaldehyde for 10 min, then washed again twice with 1X PBS. The coverslip was then inverted onto a 1.5 mm microscope slide (Globe Scientific slides 25 mm × 75 mm × 1 mm Item#: 1304 W) containing a drop of Fluoroshield with DAPI mounting media (Millipore Sigma; F6057). The slides were then imaged on a Zeiss LSM 980 confocal microscope using the 63× magnification oil lens. For the mechanistic studies, the same seeding protocol was followed. The inhibitors used were chlorpromazine (Chplr) and methyl‐β‐cyclodextrin (MBCD). GRP78 monoclonal antibody was also used to confirm the GRP78‐dependent mechanism for peptide cell uptake. After 24 h from seeding the cells, 50 nM MitoTracker was incubated with the DU145 cells for 30 min at 37°C, then washed twice with 1X PBS and chemical inhibitors chlorpromazine, at a concentration of 30 μM, methyl‐β‐cyclodextrin, at a concentration of 5 mM, were added into EMEM (1 mL) in a 1.5 mL Eppendorf tube, mixed, then added to cells containing EMEM (1 mL) for a total media volume of 2 mL. The inhibitors were incubated with the cells at 37°C for 10 min. Peptides, at a concentration of 10 μM in milli‐Q H_2_O, were then added and incubated at 37°C for an additional 30 min. The cells were then washed, fixed, and the coverslips were inverted onto a microscope slide with a drop of DAPI mounting media, and imaged. Similarly, the GRP78, at a concentration of 2 μg/μL, antibody was added to the cells and incubated for 1 h at 37°C, after MitoTracker treatment. FITC‐labelled peptides, at a concentration of 10 μM in milli‐Q water, were then added to antiGRP78‐treated cells, and allowed to incubate at 37°C for 30 min. For actin staining, stock Alexa Fluor 647 Phalloidin was diluted 1:400 in PBS and added to cells and incubated for 15 min at room temperature, then rinse once with 1× PBS. The DU145 cells were then washed twice with 1× PBS, fixed with 4% paraformaldehyde, and inverted onto a microscope slide with a drop of DAPI mounting media, and imaged by confocal microscopy. Confocal microscopy was performed with either a Zeiss LSM 980 confocal microscope with Airyscan 2 or a Nikon Ti2 microscope equipped with a Crest Xlite V3 spinning disk confocal unit. For the Zeiss microscope, images were taken on 63× power oil immersion and for the Nikon microscope, images were taken with a 60× water immersion NA 1.2 objective and a Teledyne Kinetix camera.

### 2.8 siRNA Transfections

The DU145 or WI‐38 cells were seeded in 6‐well plates at ~3.5 × 10^6^ cells per plate and incubated at 37°C for 24 h. Cells were then transfected with various concentrations (15–50 nM) of siRNA with Lipofectamine RNAiMAX (Invitrogen), used as the control industry standard transfectant reagent. The formulation was prepared by adding RNAiMAX (15 μL) to OPTIMEM (200 μL) (Gibco) with scrambled siRNA control and GRP78 silencing siRNA (5 μL, 15–50 nM, IDT – CD.Ri.389534.13.6):

siRNA Forward: AGAAUCAGAUUGGAGAUAAAGAAAA.

siRNA Reverse: UUUUCUUUAUCUCCAAUCUGAUUCUUU.

scRNA Forward: AGAUAAGCAAAGAAAGAUAUAUAGGA.

scRNA Reverse: UCCUAUAUAUCUUUCUUUGCUUAUCUUU.

to OPTIMEM (200 μL), combined and incubated for 5 min at room temperature. The mixture was then added dropwise to the cells in EMEM (2 mL), supplemented with 10% FBS, and incubated at 37°C for 48 h. Similarly, cells transfected with peptides (W1‐R9 and R9) and GRP78 silencing siRNA were also allowed to incubate for 48 h to form the ionic peptide:siRNA complex for transfection. The peptides were added at a 5:1 peptide nitrogen to siRNA phosphate (N:P) stoichiometric mole ratio [28]. Similarly, the peptides and siRNA were added separately to OPTIMEM (150 μL), and then combined, mixed and incubated at room temperature for 1 h for peptide:siRNA complex formation. Aqueous CaCl_2_ (1 M in nuclease and RNAase free water) was added to the peptide mixture in OPTIMEM (150 μL) to a stock concentration of 300 mM. An aliquot of peptide sample (20 mM CaCl_2_ in media) was added to the siRNA mixture in OPTIMEM (150 μL). Thereafter, the peptide:siRNA complex was added dropwise to the cells in EMEM (2 mL) supplemented with 10% FBS, and allowed to incubate at 37°C for 48 h. The cells were then washed three times with chilled 1× PBS and gently harvested with cell scrapers, lysed in RIPA buffer (ThermoFisher) for protein and RNA extraction in two microcentrifuge tubes by spinning samples at 5000 rpm for 10 min to pellet the cells and collect the supernatant for western blots and qRT PCR, respectively.

### 2.9 Protein Extraction and BCA Assay

The pelleted DU145 or WI‐38 cells were lysed using chilled RIPA buffer (130 μL), for 30 min in the fridge, and then briefly sonicated on a Sonics & Materials – Vibra Cell sonicator. The lysed cells were spun at 12,000 rpm at 4°C for 10 min and the supernatant was collected. Using the bicinchoninic acid (BCA, ThermoFisher) assay for protein concentration determination, protein standards were prepared from a stock of 2 mg/mL BSA in RIPA buffer. The protein standards were prepared to final concentrations (0, 0.025, 0.125, 0.25, 0.5, 0.75, 1.0, 1.5, and 2 mg/mL). A 2× dilution was prepared from all protein samples. To a 96‐well plate, 25 μL was added from each of the standards and each of the 2× protein samples in triplicate and the BCA reagent (200 μL, ThermoFisher) was added, mixed, and incubated at 37°C for 30 min in the dark. The 96‐well plate was analyzed on a (CYTATION|5 Plate Reader) plate reader with detection set at 562 nm. Data was collected, exported to Excel and the standard curve was plotted to calculate the concentration of the protein samples for each treatment condition.

### 2.10 Western Blot

The samples were mixed (1:1 v/v) with 2× Laemmli sample buffer made of 2× Laemmli buffer (BioRad) with β‐mercaptoethanol at a ratio of 50:1, respectively. The samples were incubated at 95°C for 3 min. The samples were then loaded on a 10% SDS‐PAGE starting with the Precision plus Protein All Blue Standard (BioRad) in the first lane followed by all the samples thereafter. The gel was run at 120 V for roughly 1.5 h until. Gel transfer using western blotting apparatus assembly onto an immune‐blot PVDF membrane (BioRad) that was placed on top of the gel, and enclosed in the assembly, and placed in a container filled with transfer buffer at 4°C and run overnight at 0.18 A. The following day, the membrane was removed from the western blot assembly, placed in a container, and blocked with 5% dry milk in 1× Tris‐buffered saline‐Tween 20 (TBST) for 1 h at 4°C while shaking. After blocking, the membrane was incubated with primary mouse anti‐GRP78 monoclonal antibody, mouse anti‐CHOP monoclonal antibody, unspliced rabbit anti‐XBP1 polyclonal antibody, and rabbit anti‐GAPDH monoclonal antibody or an anti‐mouse β‐actin monoclonal antibody, as loading control, in 1:1000 v/v dilution at 4°C overnight. The following day, the primary antibodies were collected for future use, and the membranes were washed for 10 min, three times with 1× TBST on a shaker at 4°C. A secondary antibody conjugated with HRP was then added to the corresponding membrane, either with an anti‐rabbit HRP conjugated antibody, or an anti‐mouse HRP conjugated antibody in at 1:5000 v/v in 5% milk in 1× TBST at 4°C for 1 h. The secondary antibodies were collected, and the membranes were washed for 10 min, three times with 1× TBST. To visualize the membranes, a Clarity Western ECL substrate Chemiluminescence (BioRad) mixture (1:1 v/v) was added to the membranes and allowed to sit at room temperature in the dark for 2 min. After which, the chemiluminescence solution was removed and the membrane was placed in a plastic sheet and imaged on the Bio‐Rad ChemiDock station to visualize the proteins. ImageLab software was used for further analysis of all western blots including protein band densitometry to determine equal protein loading using either GAPDH or β‐actin relative to treatment conditions.

### 2.11 RNA Isolation and RT qPCR

The Bio‐Rad Aurum Total RNA mini–Isolation Kit (BioRad‐ 7326820) was used for RNA isolation. All centrifugation steps were performed at room temperature (>12,000 ×*g*). Cells were collected and washed with chilled, sterile 1X PBS after siRNA transfection. The cells were lysed using cell lysis solution (RIPA buffer, 350 μL) supplemented with 1% β‐mercaptoethanol and mixed thoroughly. An aliquot of 70% ethanol (350 μL) was added and mixed. The solution (700 μL) was added to an RNA Binding Column (Aurum kit), and centrifuged. The column was removed, and the filtrate was discarded. The low stringency solution (700 μL, Aurum kit), composed of 5× Low Stringency Concentrate (diluted to 1× Low Stringency Wash) was added and centrifuged for 30 s. The Low Stringency filtrate was discarded, and the column was added back to the tube. The provided lyophilized (powder) RNase‐free DNase I (BioRad‐ 7326828) was reconstituted in of 10 mM Tris, pH 7.5 (250 μL) and mixed well. DNase I (5 μL) was diluted DNase I dilution solution (75 μL). Then, 80 μL of the diluted DNase I solution was added to the bottom of each RNA binding column and was allowed to rest for 15 min at room temperature. Directly after, 700 μL of the High Stringency Wash solution was added to each column and centrifuged for 30 s. The flow‐through filtrate was discarded and another 700 μL of Low Stringency Wash solution was added to the binding columns, centrifuged for 30 s, and the flow through was discarded once more. The columns were centrifuged for an additional 2 min to ensure the removal of any remaining wash solution. The RNA binding column was transferred to a clean microcentrifuge tube and 80 μL of elution solution was added to the membrane stack of the RNA binding columns. The samples were allowed to sit for 1 min, then centrifuged. The collected RNA samples were used directly for analysis or were stored at −20°C or −80°C for future use.

The isolated RNA samples were converted to cDNA using the Bio‐Rad iScript cDNA Synthesis Kit (Cat # ‐ 1708890). For each reaction, the following was added; 4 μL of the 5× of the iScript reaction mix, 1 μL of script Reverse Transcriptase, 100 ng–1 μg total RNA template, to a volume of 20 μL in nuclease free water. The reactions were incubated in a thermal cycler using the following protocol: Priming set for 5 min at 25°C, reverse transcription for 20 min at 46°C, reverse transcriptase inactivation for 1 min at 95°C, optional final step to hold completed reaction at 4°C. Finally, the completed cDNA samples were measured using a nanodrop (DeNovix – DS‐11 Spectrophotometer) to determine double stranded DNA concentration.

Qualitative PCR (qPCR) was performed to determine mRNA transcript levels of various biomarkers. The primers tested were GAPDH as the house keeping gene, GRP78 as the main target of interest, CHOP as apoptotic marker, and spliced XBP1 (XBP1(s)) as cell stress marker. All primers were ordered from IDT with the corresponding sequences (Table S1, ESI), including the forward and reverse primer sequences along with their NCBI references numbers. Each primer was lyophilized and reconstituted (100 μM) in nuclease free water and diluted to produce stock mixtures (10 μM). For qPCR, a Bio‐Rad SsoAdvanced Universal SYBR Green Supermix (ThermoFisher) was used for each reaction. The reactions (10 μL) were conducted as follows: 5 μL of the 2× SsoAdvanced SYBR Green, 1 μL each of the 10 μM reverse and forward primers, 100 ng cDNA diluted to 3 μL per reaction. The reactions were amplified using a BioRad CFX connect Real‐Time system qPCR machine using the manufacture's protocol.

### 2.12 Statistical Analysis

All statistical analysis was performed using GraphPad prism. The error bars for data collection of biological replicates greater than 1 (*n* ≥ 3), indicates the standard error of the mean. The statistical analysis determined to be significant (*p* < 0.05) according to a one‐tailed *t*‐test. The Fiji (ImageJ) analysis in the supplementary information was analyzed using two‐tailed *t*‐test.

## 3 Results and Discussion

### 3.1 Peptides

**FIGURE 2:**
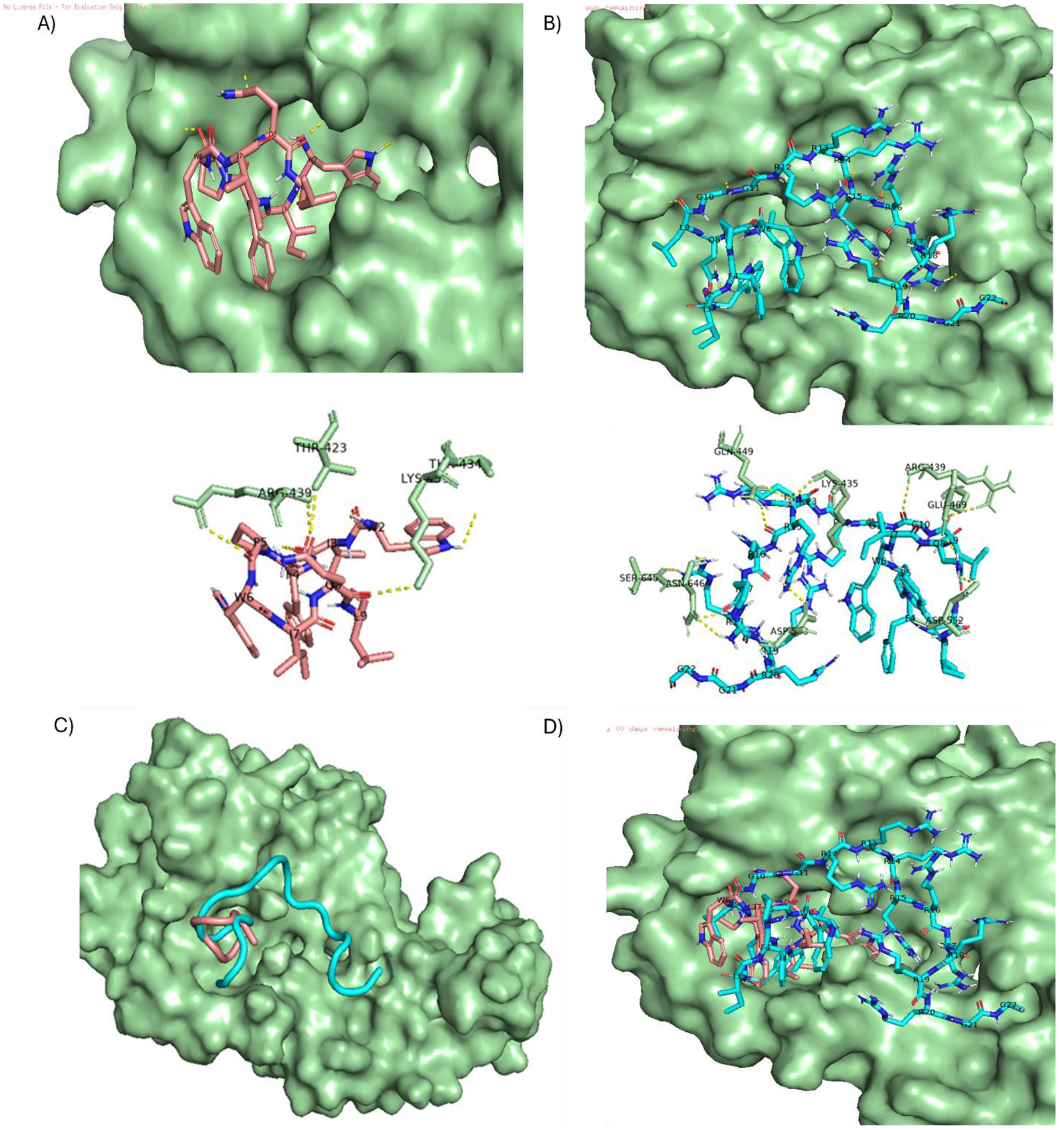
Molecular modeling and docking representation of the (A) GRP78‐targeting peptide (W1, WIFPWIQL, pink), (B) GRP78‐targeting polyarginine peptide (W1‐R9, WIFPWIQL‐R9, turquoise), (C) ribbon and (D) molecular overlay of peptides (W1, WIFPWIQL, pink) and (W1‐R9, WIFPWIQL‐R9, turquoise) within the GRP78 substrate binding domain (Thr 423 – Leu 650, PDB ID: 5E85, green). Binding (polar) interactions with peptide and GRP78 contact residues demonstrated by yellow dashed lines. Peptide (W1 – pink and W1‐R9 ‐ turquoise) and the GRP78 (substrate binding domain – green) complex generated by AutoDock and modeled in PyMOL.

Peptide synthesis and characterization were established in our previously published work [35]. The synthetic peptides (Table 1) were conjugated with fluorochrome, fluorescein isothiocyanate (FITC), linked to an aminohexanoic acid (Ahx) linker at the *N*‐terminus for peptide cell biology applications. The incorporation of Gly (‐G‐) spacers in between the CTP (W1) and CPPs (R9), and at the peptide *C*‐terminus provides spatial distance in between each peptide functional domain for GRP78 binding and cell membrane penetration directly in csGRP78‐presenting DU145 cells. The peptides incorporated biologically relevant hydrophobic and hydrophilic block segments that varied peptide polarity (hydrophobic index, HI, Table 1). The nonpolar csGRP78‐targeting CTP (W1, WIFPWIQL) (Table 1, seq. #: 1, HI: 92), binds to a hydrophobic GRP78 peptide binding pocket [32, 36]. The nona‐arginine (R9) is a polar, (Table 1, seq. #: 2, HI: 31), polycationic CPP favoring membrane permeability for intracellular gene (siRNA) delivery applications [37, 38]. The amphiphilic, arginine‐rich GRP78‐targeting peptide sequence (Table 1, seq. #: 3, W1‐R9, HI: 57), was predicted to enhance the peptide binding stability (W1 vs. W1‐R9) to the hydrophobic and anionic GRP78 peptide binding domain (Figure 2), potentially due to the additional, favorable electrostatic interactions of the polycationic CPP [32, 35, 36]. Thus, the CTP and CPP sequence compositions dictates the amphiphilic nature of the peptides, that are also anticipated to impact peptide binding activity with GRP78.

**TABLE 1:** Synthetic peptides used in this study, and their hydrophobic indices [35].

#	Peptide name^a^	Sequence^b^	Hydrophobic index^c^
1	FITC‐W1	FITC‐Ahx‐WIFPWIQL‐GG	92
2	FITC‐R9	FITC‐Ahx‐RRRRRRRRR‐GG	31
3	FITC‐W1‐R9	FITC‐Ahx‐WIFPWIQL‐GG‐RRRRRRRRR‐GG	57

^a^ Peptide abbreviation.

^b^ One‐letter amino acid sequence of synthetic peptides. Color coding matches peptide name with sequence. FITC‐labeled peptides synthesized as C‐terminal amides with G spacer and Ahx linker.

^c^ Hydrophobic index calculated by dividing the sum of hydrophobic amino acid residues by the total number of residues within the peptide sequence.

### 3.2 Molecular Modeling and Docking Studies

The crystal structures of csGRP78 revealed two main domains characterized as the substrate (peptide) binding domain (SBD) and the ATP (nucleotide) binding domain (NBD) [39, 40]. The peptide binding region of csGRP78 consists of a hydrophobic binding pocket, enabling extracellular peptide binding interactions, including with polycationic (Arg‐rich) sequences, due to the overall negative charge of GRP78 at physiological pH (7.4), which is distributed along the peptide‐binding domain [32, 36]. This conserved region displays a dynamic conformational change upon ADP binding to the ATPase region of GRP78, that closes the peptide‐binding domain for capture and release of peptide ligands upon ATP binding in the ATPase domain [32, 36]. Thus, GRP78‐targeting peptides can be leveraged for drug delivery applications. In previous studies, novel GRP78‐targeting peptide ligands have been selected by phage display bio panning, and *in‐silico* molecular modeling and docking, followed by in vitro and in vivo confirmation of peptide binding to GRP78‐presenting tumors [19, 32, 33, 34, 36]. For example, the hydrophobic GPR78‐targeting peptide (W1, WIFPWIQL) selected from phage display maintained the most favorable binding activity to csGRP78 presenting human prostate cancer (DU145) cells [19]. Molecular modeling and docking studies confirmed binding of the W1 peptide to the hydrophobic and anionic GRP78 peptide binding domain (Thr 423–Leu 650) [32, 36]. Surface plasmon resonance studies showed fast association/dissociation kinetic rate constants (k_on_/k_off_) for the W1 peptide and surface immobilized GRP78, with comparable binding activity (K_D_: ~10 μM) to related GRP78‐targeting peptide ligands [41]. However, nonpolar peptides have limited cell uptake efficiency, thereby restricting drug delivery applications.

The extension of the hydrophobic W1 peptide with the polycationic polyarginine (R9) CPP is expected to provide additional, favorable electrostatic interactions with the negatively charged residues spanning the GRP78 peptide binding domain [32, 35, 36]. A molecular modeling and docking study were implemented as part of our peptide design strategy. The binding models were used to predict the enhanced binding stability of W1‐R9 (vs. W1) to the GRP78 peptide binding domain (Figure 2). In its bound structure, non‐polar W1 peptide binds to a hydrophobic GRP78 binding pocket, while the amphiphilic W1‐R9 spans the peptide binding region by favorable hydrophobic and ionic interactions distributed along this domain (Thr 423–Leu 650) [32, 36]. Thus, the hydrophobic GRP78‐targeting W1 peptide can be functionalized with the polycationic R9 CPP to stabilize binding interactions with the hydrophobic and anionic GRP78 peptide binding domain.

### 3.3 ER Stress Induction and GRP78 Overexpression in the DU145 Cells

The human male prostate adenocarcinoma DU145 cell line has been characterized as a representative cell line with high GRP78 levels and csGRP78 presentation that confers an aggressive and resilient (castration resistant) phenotype of prostate cancer, relative to other prostate (cancer) cell lines which express little to no csGRP78 [19, 42]. Accumulating evidence also suggests that ER stress correlates with upregulation of GRP78 and enhanced translocation to the cell surface of prostate cancer cells resulting in a pro‐survival, anti‐apoptotic UPR signaling response [43, 44, 45]. Various cell stress conditions (e.g., hypoxia, cobalt chloride, thapsigargin, and tunicamycin) have been linked to GRP78 overexpression [46, 47, 48, 49]. Hypoxia is characterized by low oxygen cell incubation conditions (᷇≤ 5% O_2_), under which cells become increasingly stressed and promote stable hypoxia‐inducible factor 1α (HIF‐1α)‐mediated gene expression of hypoxia‐responsive elements (HREs), including GRP78 [46]. The cell stress inducer cobalt chloride (CoCl_2_) functions as a hypoxia mimetic by stabilizing HIF‐1α and promoting hypoxia by oxygen depletion that is also anticipated to increase GRP78 expression [47]. Thapsigargin, a stress inducer that depletes ER Ca^2+^ storage, and tunicamycin, an inhibitor of glycoprotein biosynthesis, leads to reduced protein folding by chaperone proteins, promoting ER stress induction and GRP78 overexpression [48, 49]. Therefore, various cell culture (normoxia vs hypoxia) and treatment (CoCl_2_, thapsigargin, and tunicamycin) conditions can be exploited for ER stress induction, that triggers GRP78 overexpression. Based on this evidence, the impact of ER stress on GRP78 (over)expression and cell viability in the DU145 prostate cancer cells were initially evaluated (Figure S1, ESI).

Comparison of normoxic (21% O_2_, control) with hypoxia (5% O_2_), and hypoxia inducing CoCl_2_ (100–200 μM) conditions displayed no significant changes in the GRP78 expression levels within the DU145 cells according to western blot (Figure S1A, ESI). This outcome may be potentially due to the elevated levels of GRP78 already present within the DU145 cells [50]. Similarly, a single dose (25 nM) of thapsigargin did not produce a notable increase in GRP78 expression levels. Alternatively, dose‐dependent increases (50 and 100 nM) produced a stronger ER stress response, according to the detected levels of upregulated GRP78 (Figure S1A, 17% and 26%, respectively). However, these treatment conditions resulted in a decrease in cell viability (~80%, Figure S1B, ESI) at elevated (100 nM) thapsigargin concentrations, underscoring the impact of severe ER stress on cell line toxicities. Tunicamycin treatment showed a sustained increase in detectable levels of GRP78 (66%–93%), in a concentration dependent manner (2.5–10 μg/μL) (Figure S1A, ESI), that also produced a toxic cellular response, with poor cell viability (~20%–50%, Figure S1C, ESI). While ER stress inducers can increase GRP78 levels in the DU145 cells, their significant toxicity prevents the application of these conditions in cell biology. However, considering the pronounced GRP78 expression levels detected in the absence of external stress stimuli, the DU145 prostate cancer cells were directly used as a valid and reliable cell line model for evaluating our peptide‐based GRP78‐targeting approach.

### 3.4 Peptide Cell Viability

To assess the impact of the peptides (Table 1) on cell viability, a 3‐(4,5‐dimethylthiazol‐2‐yl)‐2,5‐diphenyltetrazolium bromide (MTT) assay correlated with metabolic activity was performed with the DU145 cells over a 24‐h period (Figure S2, ESI) [51]. A peptide concentration‐dependent (0–100 μM) cell viability study was performed by the MTT assay on the DU145 prostate cancer cells under normoxic (21% O_2_, 10% FBS in EMEM at 37°C) cell culture conditions (Figure S2A, ESI). A second cell line was also selected as a negative control, displaying lower basal levels of GRP78 expression to confirm the cancer cell selectivity of our GRP78‐targeting approach. The normal (non‐tumor) human lung epithelial fibroblast WI‐38 cells were selected as a relevant control cell line due to the lower levels of GRP78 expression and its correlation with ER stress induction [52]. Moreover, the WI‐38 cells have also been validated as a negative (lower GRP78 expression levels) control cell line when compared to cancer cells with GRP78 overexpression [53]. A peptide concentration‐dependent (0–100 μM) cell viability study was also performed by the MTT assay on the WI‐38 cells (Figure S2B, ESI). In this assay, control CPP (Table 1, seq. # 2: FITC‐R9) and GRP78‐targeting peptide (Table 1, seq. # 1: FITC‐W1) were compared with the GRP78‐targeting and cell penetrating peptide (Table 1, seq. # 3: FITC‐W1‐R9). In general, the peptides maintained good (≥ 50%) cell viability, even at excess (100 μM) concentrations, and were non‐toxic at effective treatment conditions (0–10 μM) for cell biology (Figure S2, ESI). Interesting trends were also observed across the peptide sequences, with polyarginine peptides (FITC‐R9 and FITC‐W1‐R9) inducing cell proliferation at higher (25–100 μM) peptide concentrations. This outcome may be due to the activation of pro‐survival pathways leading to a higher rate of metabolism for cell division with accumulated levels of peptides [54]. Alternatively, the control GRP78‐targeting peptide (FITC‐W1) produced greater (~25%–50%) toxicity at elevated peptide concentrations (100 μM). In other reports, synthetic peptides have also displayed increased cytotoxicity at higher doses [55]. However, to determine the influence of peptide treatment on cell binding and uptake activity, biologically relevant, non‐toxic (0–10 μM) doses were selected for all peptide samples.

### 3.5 Peptide Cell Binding, Uptake, and Intracellular Localization

**FIGURE 3:**
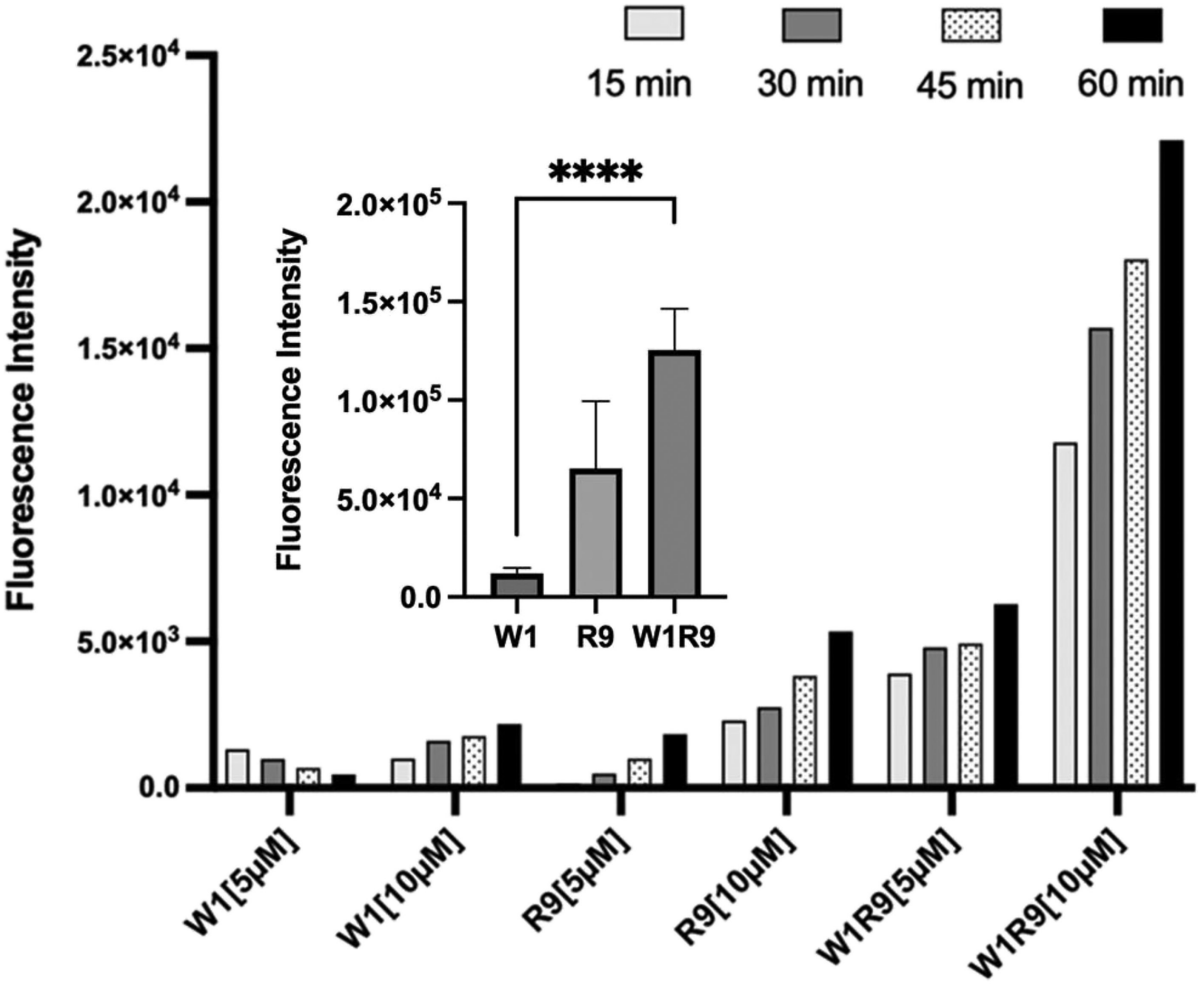
Time (0–60 min) and concentration (0–10 μM) dependent optimization of FITC‐labeled peptide binding on the DU145 cells via flow cytometry. Measurements indicating fluorescent (FITC) intensity as a function of direct peptide binding occupancy on the DU145 cells under standard (normoxia, 21% O_2_, 10% FBS in EMEM at 37°C) cell culture conditions. Inset represents fluorescence intensity measured by flow cytometry of FITC‐labeled peptides at fixed time (30 min) and concentration (10 μM) on the DU145 cells for statistical analysis and significance (one‐tailed *t*‐test *p* < 0.0001) following three independent biological replicates with the error bars representing the standard error of the mean.

Flow cytometry and confocal laser scanning fluorescence microscopy were used to monitor direct binding, cell uptake and intracellular localization of FITC‐labeled peptides within the target DU145 and control WI‐38 cells. Flow cytometry was initially used to screen peptide dose (0–10 μM) and time (0–60 min) dependent cell binding activity on the GRP78‐overexpressing DU145 prostate cancer cells (Figure 3). An increase in fluorescently (FITC‐peptide)‐labeled cells was generally observed, in a concentration and time dependent manner, with the largest population of FITC‐labeled DU145 cells detected upon peptide treatment at a concentration of 10 μM for 60 min. Interestingly, the weakest cell binding activity was observed for the GRP78‐targeting peptide (FITC‐W1) followed by cell penetrating peptide (FITC‐R9) and with superior binding observed with the combined GRP78‐targeting and cell penetrating peptide sequence (FITC‐W1‐R9). This trend suggests a cooperative peptide binding effect with the DU145 cells, for the combined GRP78‐targeting and cell penetrating peptide (CTP‐CPP:FITC‐W1‐R9) compared to the individual CTP and CPP sequences (respectively, FITC‐W1 and FITC‐R9).

**FIGURE 4:**
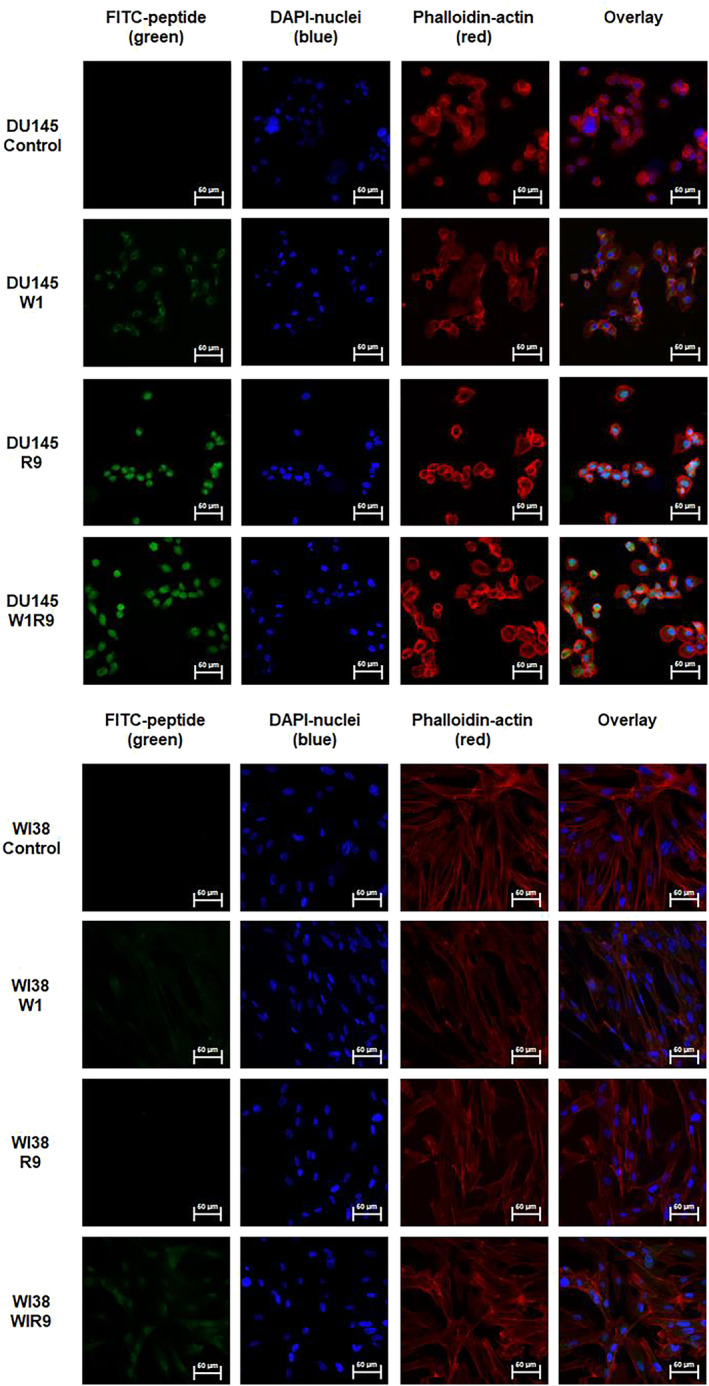
Target DU145 and control WI‐38 cells alone and treated with FITC‐labeled peptides at a concentration of 10 μM each; FITC‐W1, FITC‐R9, and FITC‐W1‐R9, for 1 h in 10% FBS EMEM at 37°C conditions. Images collected on a Zeiss LSM 980 confocal microscope using an oil lens (63×) magnification. The green corresponds to FITC peptide localization, blue corresponds to DAPI (nuclear stain), and Alexa Fluor 647 Phalloidin (actin cytoskeleton stain). Scale bars indicated are 50 μm.

To further investigate this trend, confocal fluorescence microscopy imaging revealed the impact of peptide binding activity on cell uptake (FITC‐green) and cellular localization (cytoskeleton‐actin stained with Alexa Fluor 647 Phalloidin, nuclei stained with DAPI) comparatively within the target DU145 vs. control WI‐38 cells (Figure 4). The FITC‐labeled GRP78‐targeting peptide (FITC‐W1) displayed little cell uptake in either cell line. In contrast, the CPP (FITC‐R9) exhibited noticeable cell uptake and cellular distribution in the DU145 cells and with little cell uptake detection in the WI‐38 cells. The most pronounced cell uptake in the DU145 cells was observed with the combined GRP78‐targeting and cell penetrating peptide (FITC‐W1‐R9), that was also in agreement with the enhanced cell binding activity measured by flow cytometry (Figure 3). These results confirm that the poor cell uptake capabilities of the GRP78‐targeting peptide (FITC‐W1) can be significantly improved in the presence of the CPP (R9) sequence. This result is also aligned with other reported arginine‐rich GRP78‐targeting peptide sequences [24]. Further comparative analysis of the confocal fluorescence images (Figure 4) revealed that the polyarginine functionalized GRP78‐targeting peptide (FITC‐W1‐R9) showed less cell uptake and cellular distribution in the control WI‐38 cells, with lower levels of GRP78 expression, compared to superior peptide accumulation in the GRP78 overexpressing DU145 prostate cancer cells (Figure 4). These results underscore the importance of the GRP78‐targeting cell penetrating peptide (FITC‐W1‐R9) in specific cancer cell uptake directly within GRP78‐presenting DU145 prostate cancer cells.

### 3.6 GRP78‐Dependent DU145 Cell Binding and Uptake

**FIGURE 5:**
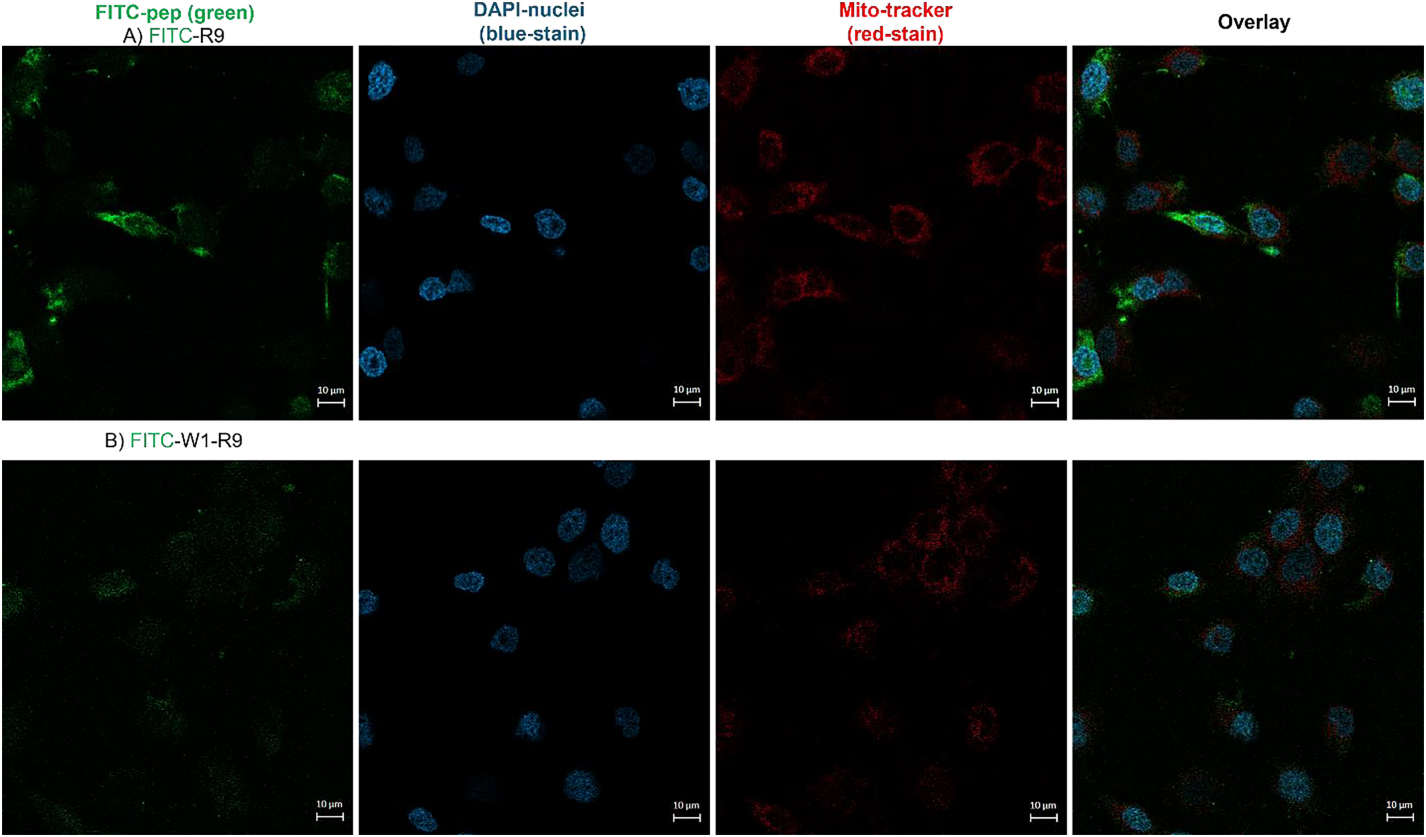
Confocal fluorescence imaging of FITC‐labeled peptide samples at a concentration of 10 μM (A) FITC‐R9 and (B) FITC‐W1‐R9 incubated at 4°C in 1% FBS with the DU145 cells. Images collected on a Zeiss LSM 980 confocal microscope using an oil lens (63×) magnification. The green corresponds to FITC‐labeled peptides (FITC‐R9 and FITC‐W1‐R9), blue corresponds to DAPI (nuclear stain), and MitoTracker Deep Red (mitochondria stain). Scale bars indicated are at 10 μm.

A mechanistic study was next conducted to test the GRP78‐dependent cell binding and uptake capabilities of the polyarginine‐derived GRP78‐targeting peptide (FITC‐W1‐R9) compared to the non‐specific CPP control (FITC‐R9). To test the influence of the polyarginine sequence on the GRP78 energy‐dependent cell uptake mechanism (Figure 5), peptide (FITC‐W1‐R9 vs. FITC‐R9) incubation under reduced serum (1% FBS) and temperature (4°C) conditions was conducted to inhibit the energy‐dependent cell uptake mechanisms of CPPs [56, 57, 58]. Serum starvation (nutrient deprivation) also serves as an “environmental stress” factor, which reduces basal cellular activity and can promote cell uptake in energy independent pathways [59]. Confocal fluorescence microscopy revealed efficient cell uptake and intracellular localization of the non‐specific polyarginine (FITC‐R9) CPP. Whereas significant inhibition of cell uptake was observed with the GRP78‐targeting polyarginine peptide (FITC‐W1‐R9), upon incubation of the DU145 prostate cancer cells under reduced serum (1% FBS) and low temperature (4°C) conditions (Figure 5 and Figure S3, ESI). This outcome suggests that the GRP78‐targeting polyarginine peptide, FITC‐W1‐R9, but not the non‐specific FITC‐R9 CPP is sensitive to an energy‐dependent cell uptake mechanism. This energy‐dependence is also associated with the reported clathrin‐receptor mediated endocytosis (CRME) mechanism of cell uptake for GRP78‐targeting peptides [19, 34]. This can be attributed to the ATP‐dependence of GRP78, with an active ATPase domain that enables the energy requirement associated with receptor mediated endocytosis of peptide binding ligands [19, 33, 34]. Thus, peptide cell uptake is inhibited at low temperature and reduced serum media, that also depletes the cellular ATP pool in an energy‐dependent cellular uptake mechanism [60]. Alternatively, under these conditions the FITC‐R9 CPP sequence can undergo cell uptake by various non‐specific and non‐energy dependent mechanisms, restricting the capacity of the R9 CPP to selectively internalize within specific cancer cell types [31].

To further examine the GRP78‐dependent mechanism of peptide cell uptake, chemical inhibitors, methyl‐β‐cyclodextrin (MβCD) and chlorpromazine (Chlpr), were tested to block internalization pathways associated with csGRP78 receptor‐mediated endocytosis [33, 34]. Chlpr inhibits clathrin‐mediated endocytosis by interfering with the formation of clathrin‐coated pits, necessary for budding of intracellular vesicles from the plasma membrane for cell uptake [61]. MβCD can also prevent the formation of clathrin‐coated endocytic vesicles by reversibly sequestering membrane cholesterol, and interfering with the invagination of caveolae lipid rafts, that inhibits cell entry [62].

**FIGURE 6:**
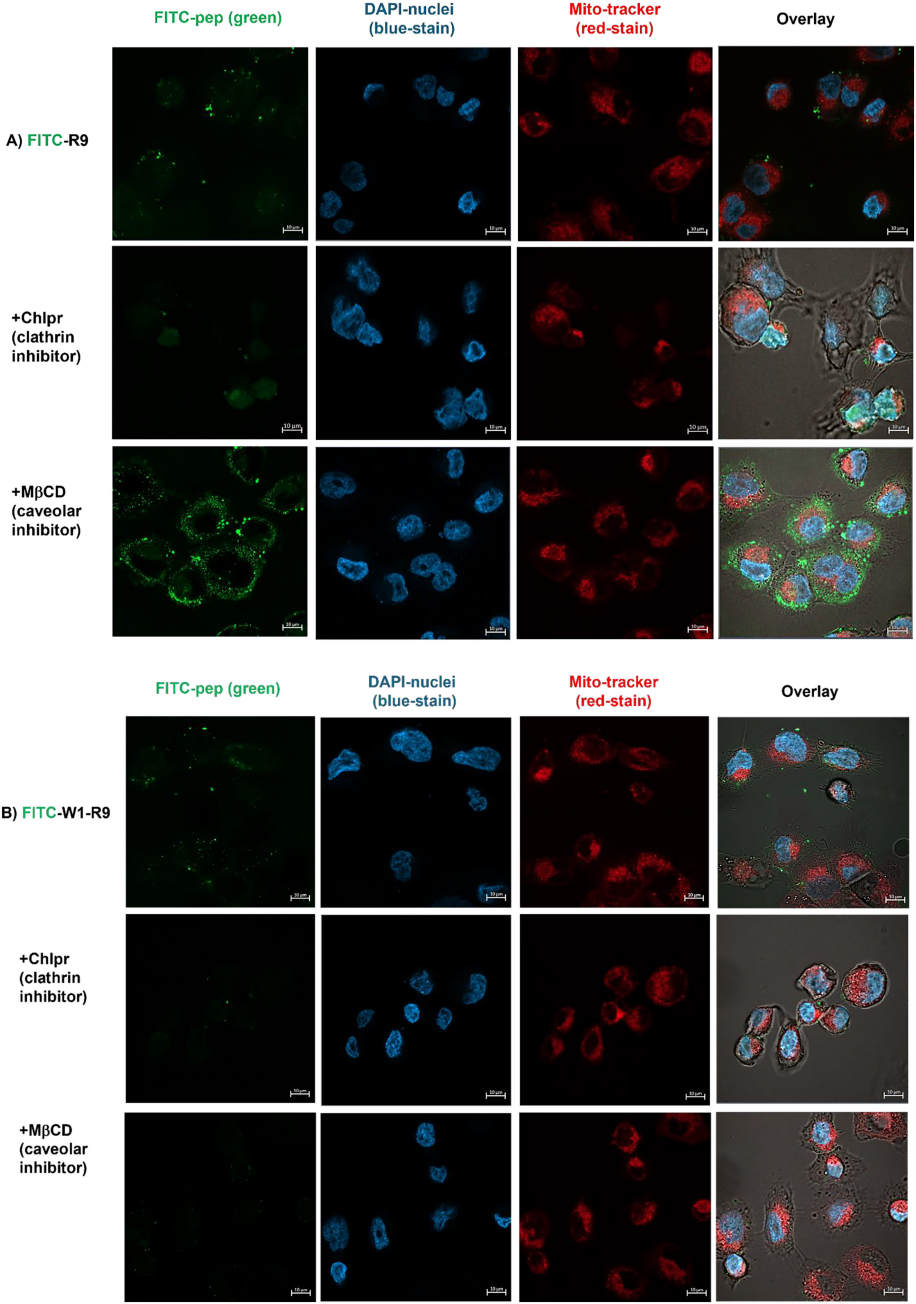
Confocal fluorescence microscopy imaging of the DU145 cells treated with FITC‐labeled peptide, at a concentration of 10 μM, (A) FITC‐R9 and (B) FITC‐W1‐R9 at 37°C in FBS free EMEM for 30 min and treated with chemical inhibitors of receptor‐mediated endocytosis. Methyl‐β‐Cyclodextrin (MβCD), at a concentration of 5 mM, for 40 min and chlorpromazine (Chlpr), at a concentration of 30 μM, for 40 min. The green corresponds to FITC‐labeled peptides (FITC‐R9 and FITC‐W1‐R9), blue corresponds to DAPI (nuclear stain), and MitoTracker Deep Red (mitochondria stain). Scale bars indicated are 10 μm.

Confocal fluorescence microscopy was used to visualize the impact of chemical inhibition on the mechanism of (csGRP78) receptor‐mediated endocytosis for peptide cell uptake (Figure 6). A direct comparison of the combined GRP78‐targeting polyarginine peptide (FITC‐W1‐R9) vs. the polyarginine CPP (FITC‐R9) with inhibitors (MβCD and Chlpr) of receptor‐mediated endocytosis was evaluated by confocal imaging (Figure 6) and quantitative analysis of the fluorescently labeled DU145 cells (Figure S4, ESI). The polyarginine CPP, (FITC‐R9) maintained cell uptake, without significant inhibition in the presence of inhibitors (MβCD and Chlpr), providing additional evidence that cell uptake for this non‐specific CPP does not depend on (csGRP78) receptor‐mediated endocytosis. Interestingly, MβCD significantly enhanced peptide FITC‐R9 cell uptake, consistent with its known mechanism for increasing cell uptake of small molecules and peptides by disruption of membrane lipid raft integrity [63]. This outcome provides further evidence that the polyarginine CPP internalizes by a variety of non‐selective cell uptake mechanisms (Figures 6A and S4, ESI) [23]. Alternatively, the combined GRP78‐targeting polyarginine peptide (FITC‐W1‐R9) displayed a significant reduction in cell uptake in the presence of MβCD and Chlpr inhibitors (Figures 6B and S4, ESI), indicating a larger dependence on clathrin‐mediated endocytosis. This mechanism may also be correlated with csGRP78 receptor peptide cell uptake for FITC‐W1‐R9 vs. the non‐specific FITC‐R9 CPP, and with pronounced cell internalization compared to the GRP78‐targeting FITC‐W1 peptide (Figures 4, S5 and S6, ESI).

**FIGURE 7:**
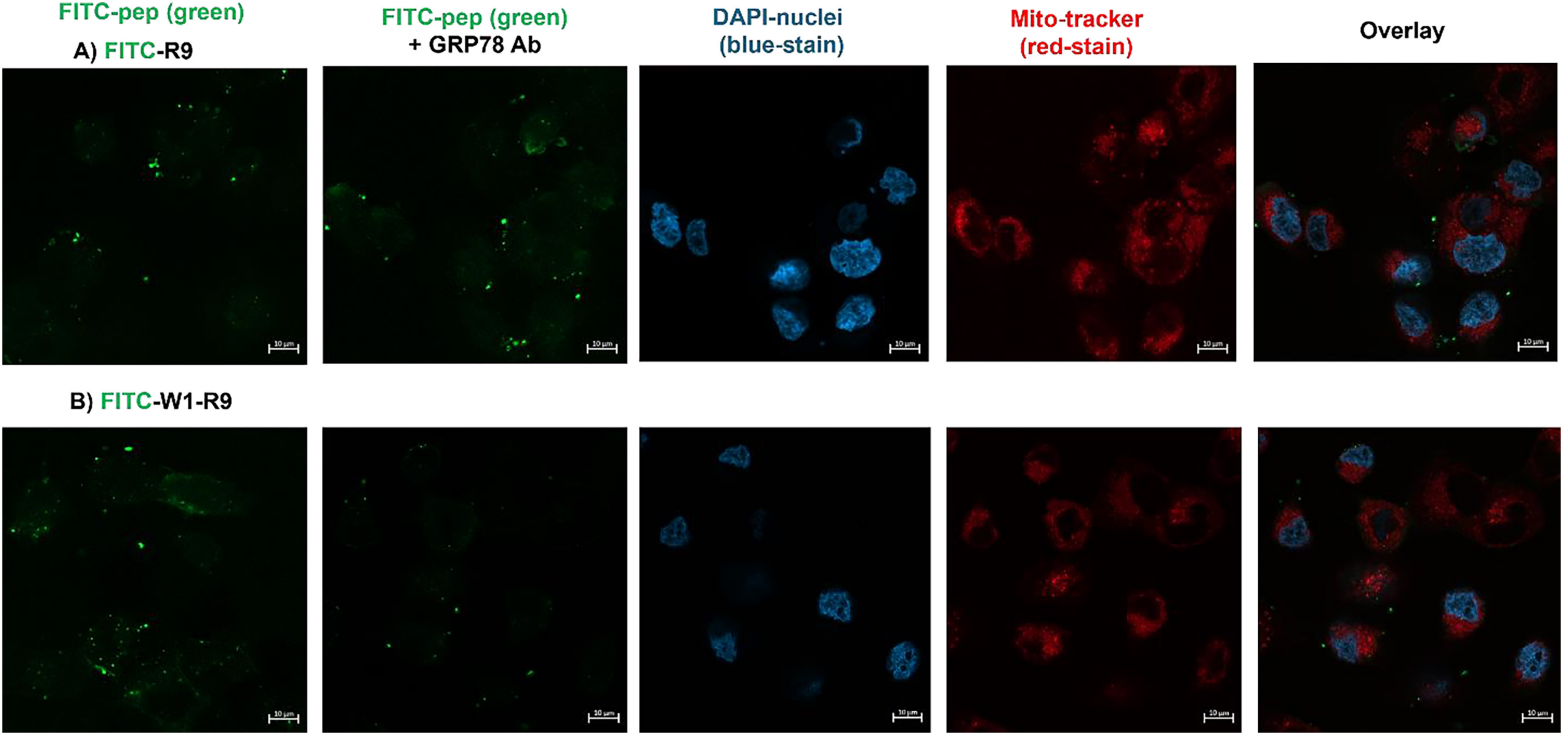
Confocal fluorescence microscopy imaging of the DU145 cells treated with FITC‐labeled peptide, at a concentration of 10 μM, (A) FITC‐R9 and (B) FITC‐W1‐R9 in the presence and absence of the primary GRP78/BiP monoclonal antibody (2 μg/mL) at 37°C in FBS free EMEM for 1.5 h. Scale bars indicated are 10 μm.

**FIGURE 8:**
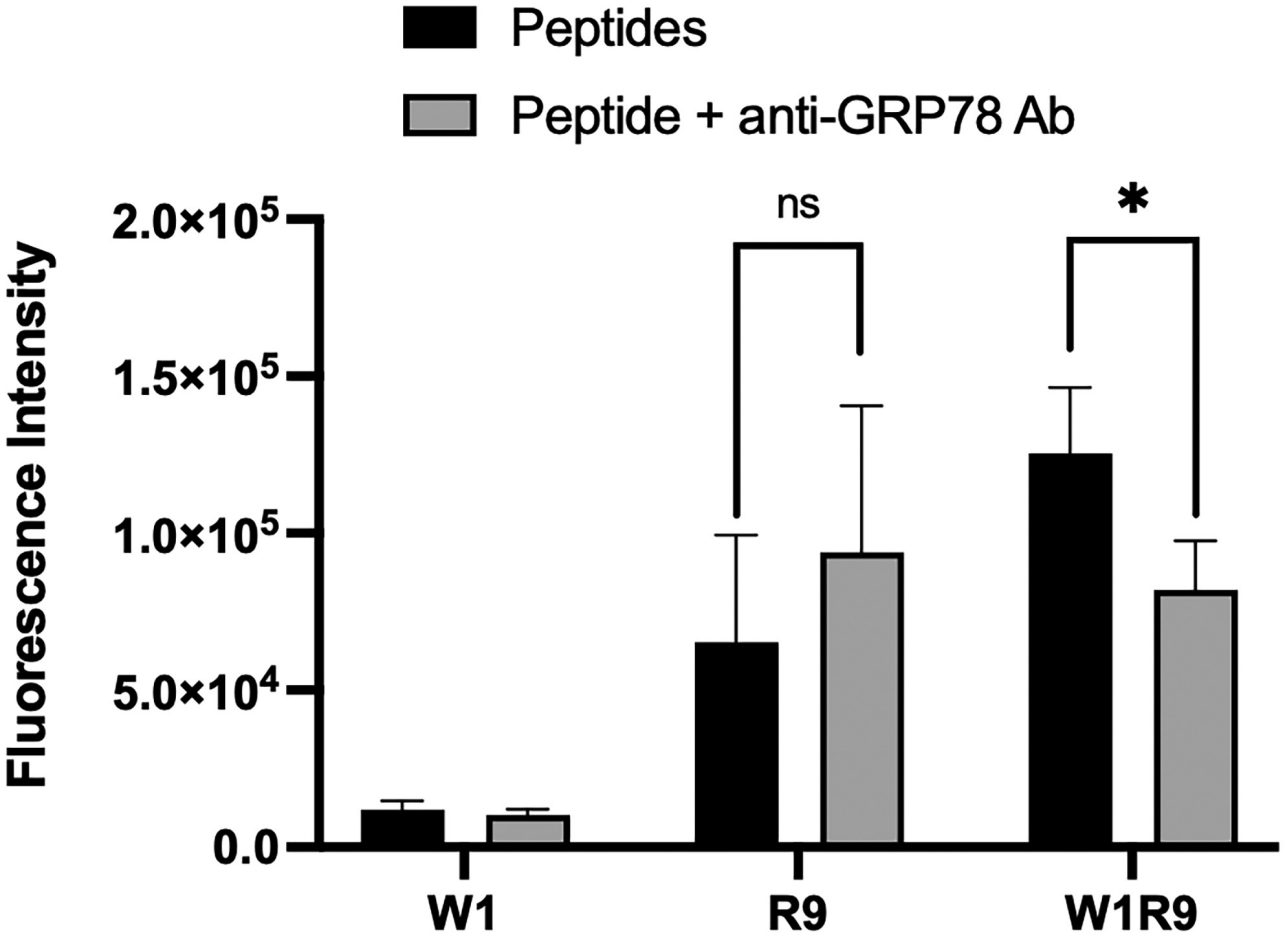
Flow cytometry binding data of FITC‐labeled peptides (10 μM, FITC‐W1, FITC‐R9 and FITC‐W1‐R9) with (black) and without (grey) the primary GRP78/BiP monoclonal antibody (2 μg/mL). The DU145 cells were treated at 37°C in FBS free EMEM. The decrease in the fluorescent intensity for W1R9 (direct vs. competitive blocking with anti‐GRP78) was found to be significant (*p* < 0.05% – with a one tailed *t*‐test) over five separate biological replicates with the error bars representing the standard error of the mean. All treatment samples were normalized against non‐treatment control condition (cells with no peptide treatment).

Confocal fluorescence microscopy (Figure 7 and Figure S7, ESI) and flow cytometry (Figure 8) were also used to confirm the GRP78‐dependent mechanism of peptide cell uptake. In competition with the primary GRP78/BiP monoclonal antibody (clone 1D6F7, ThermoFisher), selected to bind and block the peptide‐binding domain of GRP78, the binding activity of the polyarginine CPP (FITC‐R9) vs. the combined GRP78‐targeting polyarginine sequence (FITC‐W1‐R9) was examined. The FITC‐R9 CPP binds favorably (Figure 8), with apparent cell uptake in the DU145 cells (Figure 7A), with and without anti‐GRP78 inhibition. This result provides confirmation of the non‐specific, GRP78‐independent cell binding and internalization pathways associated with the FITC‐R9 CPP. In contrast, the GRP78‐targeting polyarginine peptide (FITC‐W1‐R9) displayed reduced cell binding (Figure 8) and uptake (Figure 7B) in competition with anti‐GRP78, albeit with minimal retention in cell binding and uptake due to the incorporation of the non‐specific R9 sequence. This result provides additional evidence of the GRP78‐dependent peptide cell uptake mechanism for the CTP‐CPP sequence (FITC‐W1‐R9) vs. the non‐specific CPP (FITC‐R9), and with pronounced cell internalization compared to the GRP78‐targeting (FITC‐W1) peptide alone. Therefore, the GRP78‐targeting polyarginine peptide (FITC‐W1‐R9) may serve as an efficient and selective drug (gene) delivery system directly within the csGRP78‐presenting DU145 cells.

### 3.7 Peptide:siRNA Transfection

**FIGURE 9:**
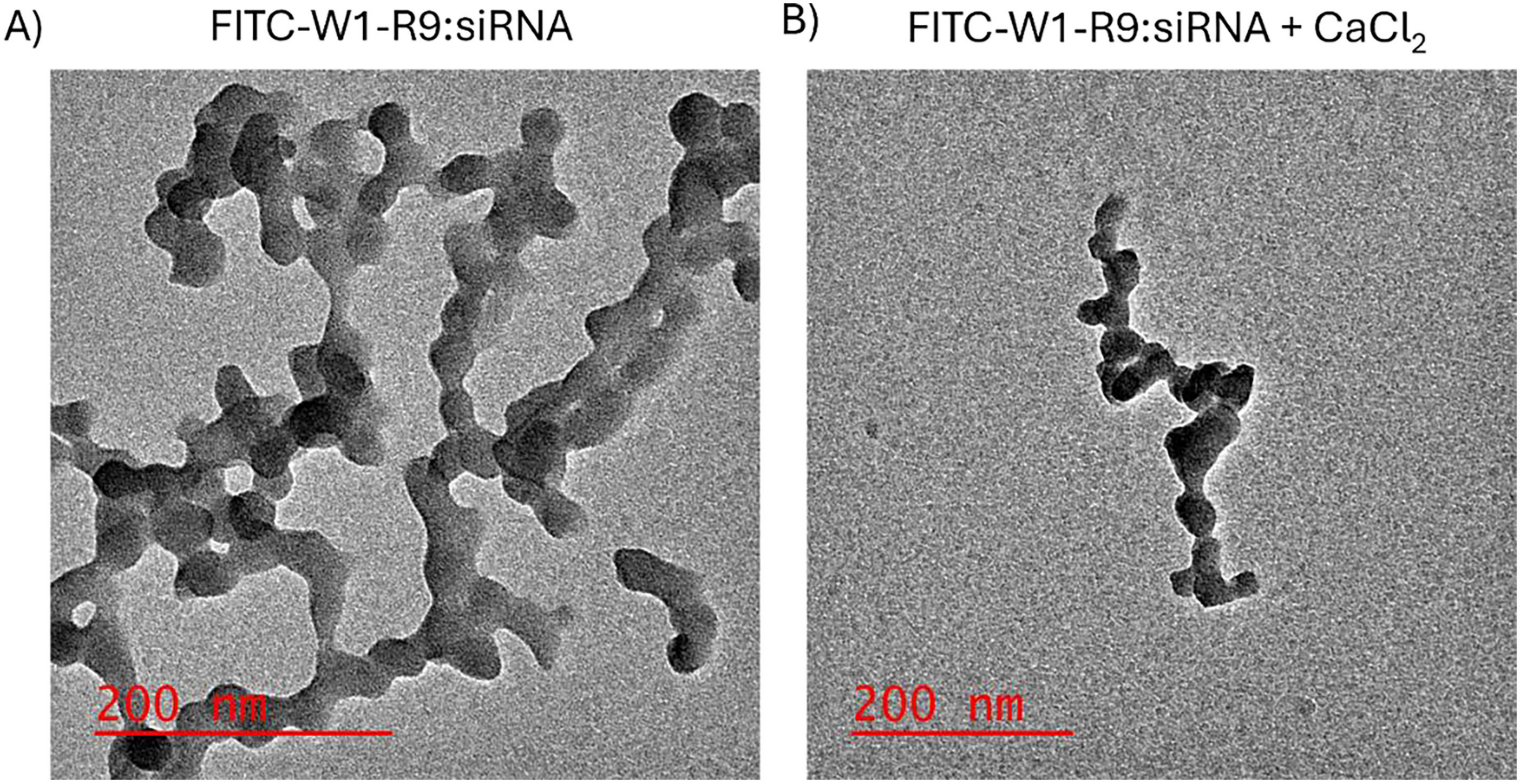
TEM images of FITC‐W1‐R9 complexed with siRNA, (A) no CaCl_2_, (B) CaCl_2_ (50 mM). Samples stained with 1% uranyl acetate in H_2_O.

A peptide‐based transfection protocol was optimized to establish a GRP78‐targeted gene (siRNA) delivery strategy for anti‐cancer applications. A peptide‐based formulation for siRNA transfection was initially developed with excess peptide:siRNA nitrogen:phosphate (5:1 N:P) stoichiometric ratios based on our previously published work, describing optimization studies for peptide:siRNA complex formation and transfection into prostate cancer cell lines [28]. The peptide:siRNA formulation formed large particle aggregates in H_2_O based on TEM imaging (Figure 9A). Interestingly, in the presence of CaCl_2_, condensed peptide:siRNA nanoparticles were observed (Figure 9B), underscoring the Ca^2+^ stabilizing effect that may also improve peptide‐based siRNA transfection efficiency [64]. The stabilizing Ca^2+^ effect on the peptide:siRNA ionic complex has also been associated with endosomal membrane destabilization, pore formation resulting in rupture and release of siRNA into the cytosol for enhancing RNAi gene silencing activity [64].

**FIGURE 10:**
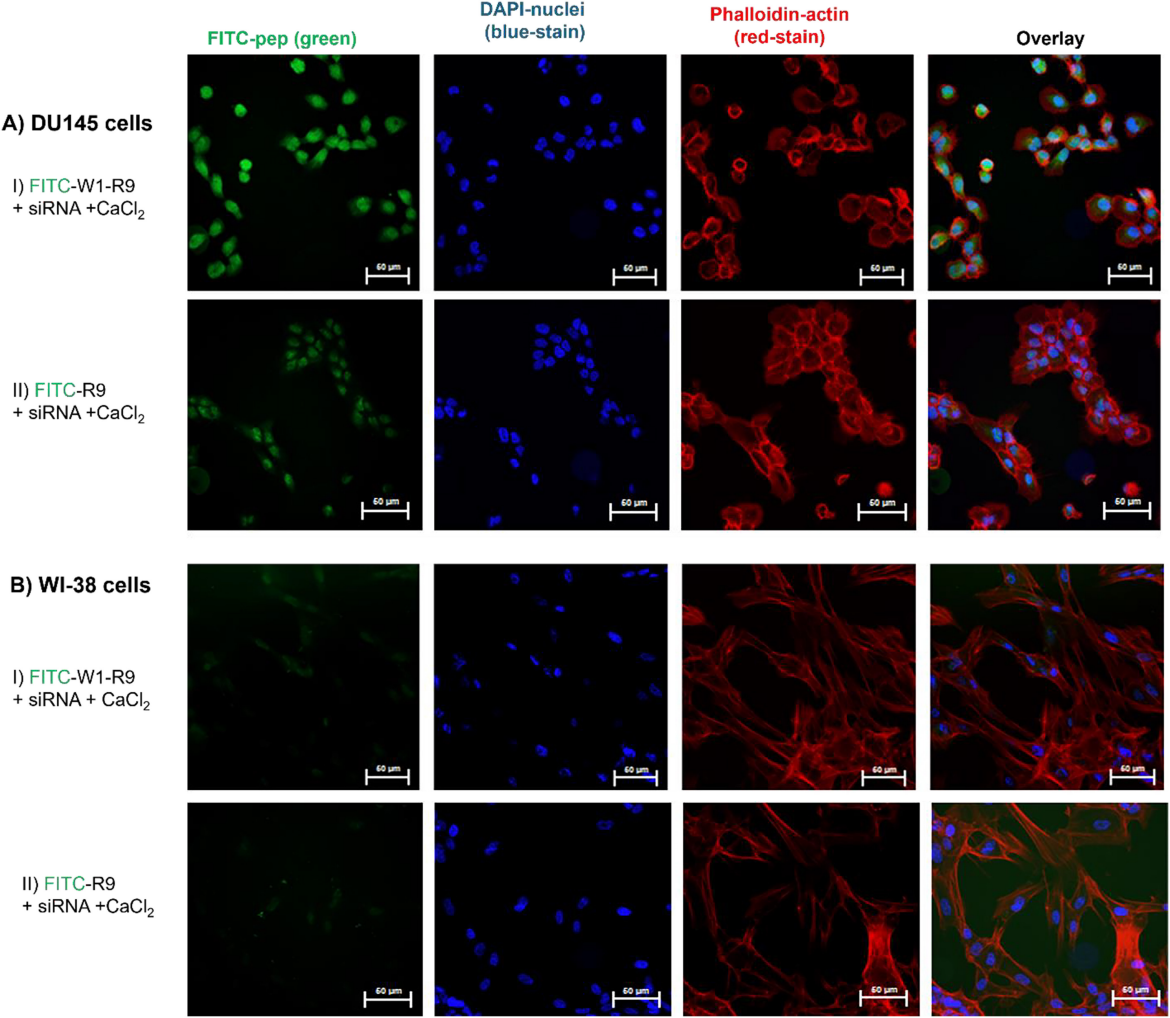
(A) Target DU145 and (B) control WI‐38 cells treated with (I) GRP78‐targeting polyarginine peptide FITC‐W1‐R9:siRNA + CaCl_2_, or (II) non‐specific CPP R9:siRNA + CaCl_2_ for 1 h in 10% FBS EMEM at 37°C conditions. Images collected on a Zeiss LSM 980 confocal microscope using an oil lens (63×) magnification. The green corresponds to FITC peptide localization, blue corresponds to DAPI (nuclear stain), and Alexa Fluor 647 Phalloidin (actin cytoskeleton stain). Scale bars are indicated at 50 μm.

A comparative assessment of the FITC‐peptide:siRNA with CaCl_2_ on cell uptake and cellular distribution within the target DU145 vs. control WI‐38 cells was performed by confocal fluorescence microscopy (Figure 10). Interestingly, the fluorochrome‐labeled GRP78‐targeting polyarginine peptide FITC‐W1‐R9:siRNA + CaCl_2_ showed minimal cell uptake and intracellular (cytosolic) localization within the control WI‐38 cells (Figure 10B), which display low levels of GRP78 expression [52, 53]. Comparatively, greater accumulation of FITC‐W1‐R9:siRNA + CaCl_2_ was detected in the DU145 cells (Figure 10A), that confer GRP78 overexpression [19, 42]. The non‐specific R9 peptide displayed less cell uptake in the DU145 cells and minimal transfection in WI‐38 cells (Figure 10), highlighting the importance of the GRP78‐targeting peptide in csGRP78‐receptor endocytosis, for specific and efficient siRNA delivery in GRP78‐presenting tumors.

Preliminary test conditions examined the impact of dose (15–50 nM) siRNA treatment on GRP78 knockdown at 48 h within the GRP78‐overexpressing DU145 prostate cancer cells (Figure S8, ESI). A direct comparison of the siRNA transfection efficiency with the peptide‐based (R9 and W1‐R9) vs. the industry‐standard RNAiMAX cationic lipid reagent indicated no significant GRP78 knockdown with the peptide:siRNA treatment conditions, whereas RNAiMAX effected noticeable (25%–35%) GRP78 silencing activity according to western blot. This outcome may be due to the poor peptide‐based siRNA transfection efficiency that is associated with the large, aggregated particles (Figure 9A) that inhibits cell uptake or endosomal escape [28]. To improve peptide:siRNA transfection efficiency, CaCl_2_ was added to condense the peptide:siRNA formulation into stable nanoparticles (Figure 9B) that was amenable to cell uptake (Figure 10A). Furthermore, CaCl_2_ has also been reported to improve the endosomal escape capacity of siRNA for enhanced gene silencing (RNAi) activity [64]. The addition of CaCl_2_, at a concentration of 20 mM, enhanced the peptide‐based siRNA transfection efficacy in the DU145 cells (Figures S9 and S10, ESI), resulting in noticeable (~50%–60%) GRP78 knockdown at the protein levels of expression based on western blot (Figure S10, ESI). A comparable reduction in *grp78* mRNA transcript levels (Figure S9, ESI) was also observed according to quantitative reverse transcription‐polymerase chain reaction (qRT‐PCR), that was comparable to the benchmark RNAiMAX transfection conditions. Less GRP78 knockdown was observed at the mRNA transcript and protein levels of expression with the GRP78‐targeting W1‐R9:siRNA + CaCl_2_ transfection conditions in the control WI‐38 cells, compared to the more pronounced GRP78 knockdown effect observed in the DU145 cells (Figures S9 and S10, ESI). These results confirm the GRP78‐dependence of the tumor (DU145) vs. non‐tumor (WI‐38) cells. The GRP78‐targeting peptide:siRNA transfection specificity may also serve to sensitize the DU145 cells towards a therapeutic anti‐cancer effect.

**FIGURE 11:**
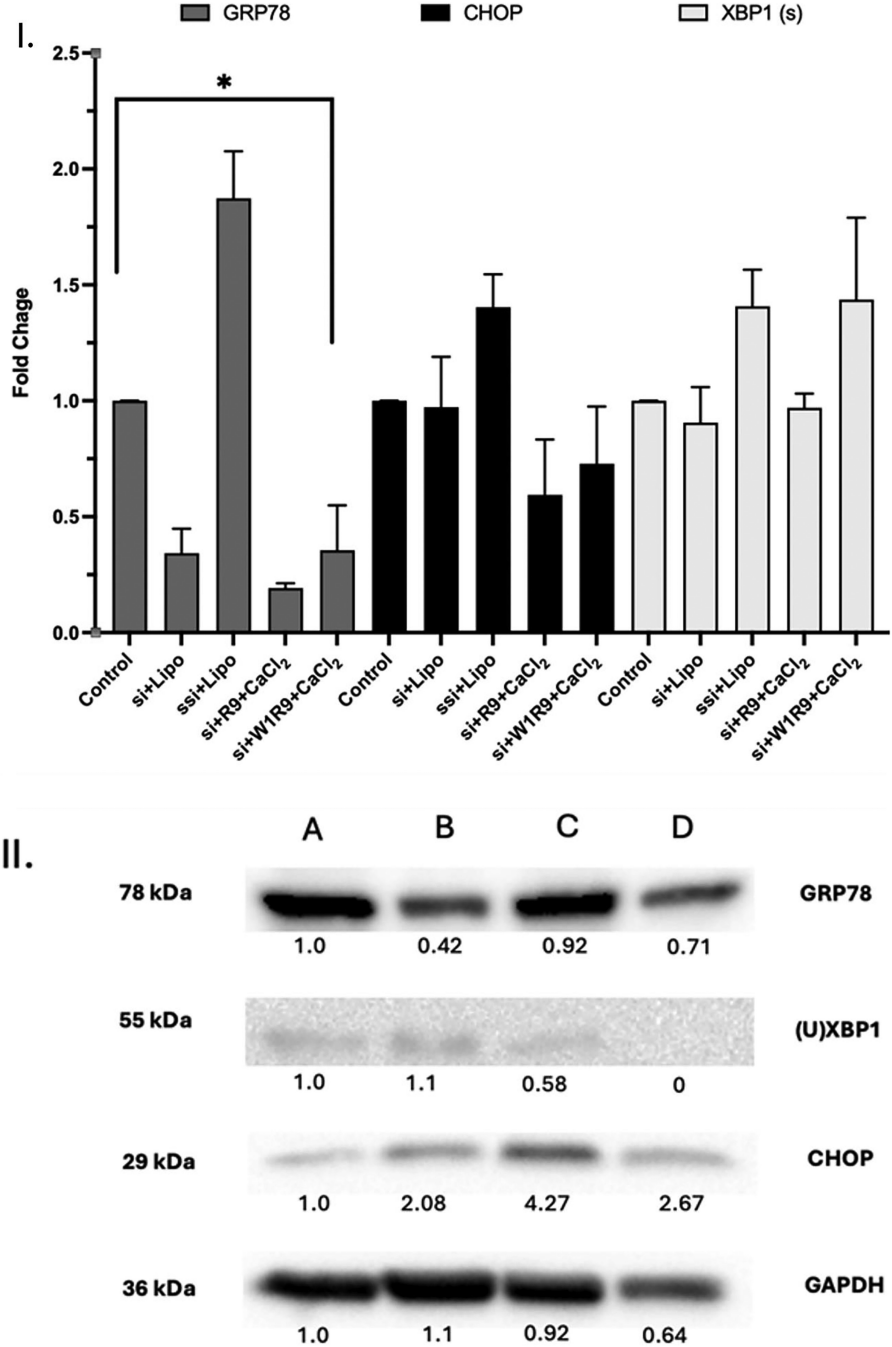
GRP78 knockdown in the DU145 cells. Peptide:siRNA transfection at 5:1 N:P molar ratio. DU145 cells transfected with GRP78 siRNA over a 48‐h period in EMEM supplemented with 10% FBS. (I) qRT‐PCR data and (II) western blot of GAPDH (control housekeeping gene), GRP78, spliced and unspliced (s/u) XBP1 as ER stress markers, in addition to CHOP as apoptotic marker. Transfection conditions: (A) control (no treatment), (B) RNAiMAX (15 μL) + siRNA at 25 nM, (C) W1R9 + siRNA at 25 nM, (D) W1R9 + siRNA at 25 nM + CaCl_2_ at 20 mM. Additional control conditions for qRT‐PCR include: RNAiMAX Lipofectamine + scrambled siRNA at 25 nM, and siRNA+R9 + CaCl_2_ at 20 mM. Statistical analysis and significance (one‐tailed *t*‐test *p* < 0.0001) based on three independent biological replicates with the error bars representing the standard error of the mean.

The optimized peptide‐based transfection conditions were used to examine the impact (if any) of GRP78 silencing on ER stress (XBP1u/s) and apoptosis (CHOP) markers associated with cancer biology (Figure 11). Interestingly, *grp78* mRNA silencing resulted in a general increase of ER stress (spliced XBP1(s)) and maintenance of the apoptotic (CHOP) markers, compared to control (no treatment) conditions, suggesting an interplay in between pro‐survival and cell death outcomes under ER stress induction (Figure 11, I) [9]. This gene regulatory effect is also associated with the observed protein levels of expression (Figure 11, II), that upon GRP78 silencing, elevated ER stress, because of diminished unspliced XBP1(u) expression levels, resulted in the upregulation of the pro‐apoptotic CHOP marker in the DU145 cells. This result correlates with the effects of sustained, late‐stage ER stress induction on the programmed cell death response [6]. Moreover, a direct correlation (cross‐talk) is evident with the increasing spliced XBP1(s) transcript levels and the decreasing unspliced XBP1(u) protein levels of expression, occurring to the most significant extent with the optimized GRP78‐targeting peptide‐based (W1‐R9) siRNA transfection conditions using CaCl_2_ (Figure 11). The downregulation of GRP78 activity within the DU145 prostate cancer cells that display an addiction to GRP78 overexpression suggests a molecular interplay in between the pro‐survival and apoptotic executors in the highly stressed (DU145) tumor microenvironment [7]. Therefore, targeting csGRP78 with GRP78‐targeting polyarginine peptides enables efficient and selective cell uptake for siRNA delivery and silencing activity promoting anti‐cancer effects within the resilient GRP78‐overexpressing DU145 prostate cancer cells.

The ER stress‐inducible chaperone, GRP78, acts as a master regulator of UPR biomarkers that are responsible for the maintenance of cell homeostasis, pro‐survival and apoptotic effects [2, 3, 4, 5, 6]. During ER stress, via accumulation of misfolded proteins, GRP78 is released from the main UPR sensors, inositol‐requiring kinase 1 (IRE1), protein kinase R‐like ER kinase (PERK), and activating transcription factor 6 (ATF6), to initiate the UPR signaling pathway [4]. Upon GRP78 dissociation, PERK dimerization results in activation via autophosphorylation and phosphorylates the eukaryotic initiation factor 2 alpha (eIF2α), which further phosphorylates eIF2β, leading to the reduction of protein load by the inhibition of protein synthesis [7, 8, 65]. Similarly, IRE1 is activated, enabling the splicing of the unspliced mRNA transcript of X‐box binding protein 1 (XBP1(u)), into its spliced XBP1(s) form [66, 67]. Mature XBP1(s) functions as a transcription factor that translocates to the nucleus to induce gene expression of ER‐associated protein degradation (ERAD), which mitigates cellular damage caused by the accumulation of misfolded proteins [67, 68]. The UPR stress element, ATF6 is also dissociated from GRP78 under ER stress and translocated to the Golgi, where it is cleaved and transported to the nucleus acting as a transcription factor [69]. Nuclear ATF6, activates gene expression of chaperone proteins, such as GRP78, that assist in ER protein folding to resolve ER stress and maintain cellular homeostasis [70]. In this study, the pronounced reduction in *grp78* mRNA transcript levels detected by qRT‐PCR (Figure 11, I), resulted in an overall induction in ER stress (spliced XBP1(s)) and regulation of apoptotic (CHOP) markers, suggesting an initial interplay in between survival and apoptotic cellular stress responses.

Alternatively, during excessive and prolonged ER stress insult, GRP78 overexpression can result in UPR‐dependent programmed cell death (apoptosis). This stress‐induced cellular response activates downstream UPR markers of PERK and ATF6, C/EBP‐homologous protein or DNA damage‐inducible transcript 3 (CHOP), by translation of activating transcription factor 4 (ATF4) via phosphorylation of eIF2α, ultimately resulting in cell apoptosis [7, 71]. In this signaling pathway, ATF4 can upregulate CHOP expression, which plays a pivotal role in the apoptotic response by the upregulation of ER oxidoreductin 1 (ERO1), involved in oxidative protein folding resulting in cell death due to ROS accumulation [71]. Furthermore, CHOP induction via pronounced ER stress can lead to the activation of pro‐apoptotic proteins (BAX, BIK), the release of cytochrome *c*, and accumulated levels of cleaved‐caspases 3, 7, 8, and 12 that promote apoptosis [72]. Interestingly, this study revealed that upon GRP78 silencing, an elevated ER stress response was detected, based on diminished unspliced XBP1(u) protein expression levels, that resulted in the upregulation of the pro‐apoptotic CHOP marker according to western blot (Figure 11, II). This result correlates with the pro‐apoptotic responses from sustained, late‐stage ER stress induced apoptosis [72]. This outcome is also supported by the increasing spliced XBP1(s) transcript levels and decreasing unspliced XBP1(u) protein levels of expression, following GRP78 knockdown with the optimized peptide‐based (W1‐R9) siRNA transfection conditions using CaCl_2_ (Figure 11). A direct correlation with ER stress‐induced cell death is also apparent with the downstream pro‐apoptotic signaling marker CHOP. Thus, the downregulation of GRP78 expression in the DU145 prostate cancer cells results in a molecular interplay in between ER stress and apoptotic responses that hinge on GRP78 activity [2].

Unlike GRP78 knockout or gene edited cell lines [73], partial and/or transient GRP78 downregulation enables this peptide‐based GRP78‐targeting strategy to be applicable in the shorter to longer‐term, for the evaluation of GRP78 silencing activity on cancer biology. Interestingly, less GRP78 expression and knockdown activity was observed at the mRNA transcript and protein levels of expression in the WI‐38 cells, compared to a more pronounced GRP78 overexpression and silencing effect in the DU145 cells (Figures S9 and S10, ESI). This outcome is consistent with the GRP78 dependence of the DU145 prostate cancer cells compared to the normal, non‐tumorigenic WI‐38 cells that display lower, basal levels of GRP78 expression [42, 74]. The detected mRNA vs. protein levels of GRP78 expression are also correlated with the RNAi mechanism of siRNA gene silencing activity. A direct impact on mRNA transcript levels, and with later, variable effects observed at the protein levels of expression that may depend on extended protein turnover rates, metabolism and post‐transcriptional/translational modifications and regulation [43, 75]. For example, GRP78 protein knockdown produced noticeable induction of ER stress (XBP1u/s) and apoptosis (CHOP) markers regulating cancer cell survival and death in the resilient, castration‐resistant prostate cancer (DU145) cell line (Figure 11) [76]. Therefore, this specific peptide‐based GRP78‐targeting strategy is applicable to siRNA delivery for the evaluation of GRP78 silencing activity on cancer biology.

## 4 Conclusions

In conclusion, this research demonstrated the potential for using GRP78‐targeting polyarginine peptides for gene (siRNA) delivery. A new set of synthetic fluorescein‐labelled amphiphilic peptides composed of csGRP78 targeting and arginine‐rich cell penetrating domains were designed and developed for investigating cancer biology. The peptides displayed efficient GRP78‐dependent cell binding and uptake in a representative human prostate cancer (DU145) cell line with elevated GRP78 levels. Mechanistic studies revealed an energy‐dependent, GRP78‐receptor mediated endocytosis mechanism for cell uptake of the GRP78‐targeting polyarginine peptide (W1‐R9). The detected cytosol accumulation of this peptide underscored its potential utility in siRNA delivery. The GRP78‐targeting peptide:siRNA complexes formed stably condensed ionic nanoparticles with calcium chloride (CaCl_2_) functioning as ionic stabilizer and additive that improved peptide‐based (siRNA) transfection efficiency and specificity in tumor (DU145) vs. non‐tumor (WI‐38) cells. Peptide‐based siRNA transfection studies in the DU145 cells indicated that GRP78 knockdown led to an interplay in between ER stress, pro‐survival and cell death responses that potentiate anti‐cancer effects. Thus, the GRP78‐targeting polyarginine peptides are also anticipated to function as efficient therapeutic gene delivery systems directly in GRP78‐presenting tumors, for applications related to cancer‐targeted gene therapy. Considering cell surface GRP78 is found in cancer but absent or minimally expressed on the surface of non‐malignant tissues [16, 17], this GRP78‐targeting approach may broadly function as a therapeutic gene (e.g., siRNA, mRNA and pDNA) delivery system across various GRP78‐presenting tumors [77]. This precision oncology strategy may also circumvent the toxic, non‐selective drug delivery (e.g., lipid‐based) formulations and conventional cancer therapy strategies that cause severe side‐effects, raise the risk of tumor recurrence and metastatic spread from therapy resistance [78]. Therefore, tumor‐homing and penetrating peptides offer great potential as the next‐generation precision medicines in oncology.

## Author Contributions

G.H., G.D., F.C., conducted experimental investigations. W.G.W., and D.S., supervised, secured project funding, and compiled the manuscript. All authors have read and approve this manuscript.

## Conflicts of Interest

The authors declare no conflicts of interest.

## Supporting information


**Figure S1.** (A) Western blot of the GRP78 expression levels within the DU145 cells under control (normoxia) and ER stress conditions. The cells were incubated under normoxia (21% O_2_) or hypoxia (5% O_2_), or increasing concentrations of CoCl_2_ [100, 150, & 200 μM], thapsigargin [25, 50, 100 nM], or tunicamycin [2.5, 5, 10 μg/mL] treatment for 24 h in 10% FBS EMEM at 37°C. MTT cell viability assay for DU145 cells treated with (B) thapsigargin [25, 50, 100 nM] and (C) tunicamycin [2.5, 5, 10 μg/mL] for 24 h in 10% FBS EMEM at 37°C. Graphpad prism was used to generate the plots and analyze all statistical data.
**Figure S2.** MTT cell viability assay of peptides at various concentrations [0, 10, 25, 50, 75 and 100 μM] tested on the (A) DU145 prostate cancer cells and (B) WI‐38 lung fibroblast cells for 24 h of incubation time in 10% FBS in EMEM at 37°C. The error bars shown reflect the standard error of the mean. Graphpad prism was used to generate the plots and analyze all statistical data.
**Figure S3.** The corrected total cell fluorescence (CTCF) as a quantitative measurement of the fluorescence intensity of the FITC‐labeled peptides (FITC‐R9 and FITC‐W1‐R9) incubated at 4°C in 1% FBS with the DU145 cells. Data extrapolated from the confocal imaging (Figure 5) using Zen blue software. Statistical analysis and significance (two‐tailed *t*‐test *p* < 0.0001) was accomplished by analyzing cells within each treatment using Fiji (ImageJ) and averaged, where the error bars reflect the standard error of the mean, showing significant decrease in fluorescence intensity for W1R9 relative to R9 peptide. Graphpad prism was used to generate the graph and analyze all statistical data.
**Figure S4.** The corrected total cell fluorescence (CTCF) as a quantitative measurement of the fluorescence intensity of the FITC‐labeled peptides (FITC‐R9 and FITC‐W1‐R9) with the DU145 cells incubated with chemical inhibitors (chlorpromazine and methyl‐β‐cyclodextrin) of receptor‐meditated endocytosis. Data extrapolated from the confocal imaging (Figure 6) using Zen blue software. Statistical analysis and significance (two‐tailed *t*‐test *p* < 0.001 and 0.0001, respectively) was accomplished by analyzing cells within each treatment using Fiji (ImageJ) and averaged, where the error bars reflect the standard error of the mean. Graphpad prism was used to generate the graph and analyze all statistical data.
**Figure S5.** DU145 cells treated with (10 μM) W1 peptide at 37°C in FBS free EMEM for 30 min, in addition to various receptor mediated endocytosis inhibitors to determine peptide uptake mechanism. (A) is treated with W1 peptide alone as a baseline control, (B) treated with [5 mM] Methyl‐β‐Cyclodextrin for 40 min, (C) treated with (30 μM) Chlorpromazine for 40 min.
**Figure S6.** The corrected total cell fluorescence (CTCF) as a quantitative measurement of the fluorescence intensity of the targeting FITC‐W1 peptide in the DU145 cells. Data extrapolated from the confocal imaging (Figure S5) using Zen blue software. Statistical analysis showed no significance amongst the different treatments. Cell analysis within each treatment was done using Fiji (ImageJ) and averaged, where the error bars reflect the standard error of the mean. Graphpad prism was used to generate the graph and analyze all statistical data.
**Figure S7.** The corrected total cell fluorescence (CTCF) as a quantitative measurement of the fluorescence intensity of the FITC‐labeled peptides (FITC‐R9 and FITC‐W1‐R9) with the DU145 cells incubated with anti‐GRP78. Data extrapolated from the confocal imaging (Figure 7) using Zen blue software. Statistical analysis and significance (two‐tailed *t*‐test *p* < 0.01) was accomplished by analyzing different cells within each treatment using Fiji (ImageJ) and averaged, where the error bars reflect the standard error of the mean. Graphpad prism was used to generate the graph and analyze all statistical data.
**Figure S8.** Transfection study in the DU145 cells showing GRP78 protein downregulation with various siRNA concentrations in combination with RNAiMAX as the industry standard control, in comparison with cell penetrating peptide (R9) and the cell targeting and penetrating peptide W1‐R9. Western blot of GRP78 normalized against GAPDH as loading control values. Transfection conditions include: (A) control, no treatment (B) RNAiMAX + siRNA [15 nM], (C) RNAiMAX + siRNA [25 nM], (D) RNAiMAX + siRNA [50 nM], (E) R9 + siRNA [25 nM], (F) R9 + siRNA [50 nM], (G) W1R9 + siRNA [25 nM], (H) R9 + siRNA [50 nM]. Peptides:siRNA were combined in a 5:1 N:P molar ratio.
**Figure S9.** Quantitative qRT PCR data relating the downregulation of GRP78 expression at the mRNA transcript levels. The peptides were incubated at [5:1 N:P] mole ratio relative to the siRNA. For transfections, the DU145 and WI‐38 cells were incubated for 48 h showing (from left to right): Control (no treatment), siRNA [25 nM] + RNAiMAX Lipofectamine, scrambled siRNA (ssi, [25 nM]) + RNAiMAX Lipofectamin, siRNA + R9 + CaCl_2_ [20 mM], and siRNA + W1R9 + CaCl_2_ [20 mM]. Statistical analysis and significance (two‐tailed *t*‐test *p* < 0.05) was accomplished by analyzing different cells within each treatment using Fiji (ImageJ) and averaged, where the error bars reflect the standard error of the mean. Graphpad prism was used to generate the graph and analyze all statistical data.
**Figure S10.** Western blot relating the downregulation of GRP78 expression at the protein levels. The peptides were incubated at [5:1 N:P] mole ratio relative to the siRNA. For transfections, the DU145 or WI‐38 cells were incubated for 48 h with: (A) control, no treatment (B) RNAiMAX Lipofectamine (15 μL) + siRNA [25 nM], (C) siRNA + CaCl_2_ [20 mM], (D) siRNA+R9 + CaCl2 and (E) siRNA + W1R9 + CaCl_2_.
**Table S1.** List of sense and antisense primer sequences for qRT PCR from Integrated DNA Technologies (IDT). The NM shows the reference sequence number taken from NCBI.

## Data Availability

The data that support the findings of this study are available from the corresponding author upon reasonable request.
