# Supplementary material for: Cancer‐Targeting Peptides Functionalized With Polyarginine Enables GRP78‐Dependent Cell Uptake and siRNA Delivery Within the DU145 Prostate Cancer Cells
Source: J Pept Sci. 2025 Feb 18;31(3):e70007. doi: 10.1002/psc.70007 (PMC11836551; doi:10.1002/psc.70007)
Supplement: Supplementary file 1 — Figure S1. (A) Western blot of the GRP78 expression levels within the DU145 cells under control (normoxia) and ER stress conditions. The cells were incubated under normoxia (21% O2) or hypoxia (5% O2), or increasing concentrations of CoCl2 [100, 150, & 200 μM], thapsigargin [25, 50, 100 nM], or tunicamycin [2.5, 5, 10 μg/mL] treatment for 24 h in 10% FBS EMEM at 37°C. MTT cell viability assay for DU145 cells treated with (B) thapsigargin [25, 50, 100 nM] and (C) tunicamycin [2.5, 5, 10 μg/mL] for 24 h in 10% FBS EMEM at 37°C. Graphpad prism was used to generate the plots and analyze all statistical data. Figure S2. MTT cell viability assay of peptides at various concentrations [0, 10, 25, 50, 75 and 100 μM] tested on the (A) DU145 prostate cancer cells and (B) WI‐38 lung fibroblast cells for 24 h of incubation time in 10% FBS in EMEM at 37°C. The error bars shown reflect the standard error of the mean. Graphpad prism was used to generate the plots and analyze all statistical data. Figure S3. The corrected total cell fluorescence (CTCF) as a quantitative measurement of the fluorescence intensity of the FITC‐labeled peptides (FITC‐R9 and FITC‐W1‐R9) incubated at 4°C in 1% FBS with the DU145 cells. Data extrapolated from the confocal imaging (Figure 5) using Zen blue software. Statistical analysis and significance (two‐tailed t‐test p < 0.0001) was accomplished by analyzing cells within each treatment using Fiji (ImageJ) and averaged, where the error bars reflect the standard error of the mean, showing significant decrease in fluorescence intensity for W1R9 relative to R9 peptide. Graphpad prism was used to generate the graph and analyze all statistical data. Figure S4. The corrected total cell fluorescence (CTCF) as a quantitative measurement of the fluorescence intensity of the FITC‐labeled peptides (FITC‐R9 and FITC‐W1‐R9) with the DU145 cells incubated with chemical inhibitors (chlorpromazine and methyl‐β‐cyclodextrin) of receptor‐meditated endocytosis. Data extrap [file PSC-31-e70007-s001.docx]

**Electronic Supplementary Information^†^**

Cancer-Targeting Peptides Functionalized with Polyarginine Enables GRP78-Dependent Cell Uptake and siRNA Delivery within the DU145 Prostate Cancer Cells

George Hilan,^1^ Grace Daniel,^2,3^ Filiz Collak,^1,2^ David Sabatino,^2,3,^* and William G. Willmore^1,2,3,^*

^1^Department of Biology, ^2^Department of Chemistry, and ^3^Institute of Biochemistry, Carleton University, 1125 Colonel By Drive, Ottawa ON, K1S 5B6, Canada

*Corresponding authors.

e-mail: [david.sabatino@carleton.ca](mailto:david.sabatino@carleton.ca) and [Bill.Willmore@carleton.ca](mailto:Bill.Willmore@carleton.ca)

**Table of Contents**

**Fig. S1.** Western blot and cell viability assay under ER stress………………………………...S2

**Fig. S2.** Cell viability (MTT) assay with peptides...…………………………………………...S3

**Fig. S3.** Quantitative fluorescence for peptides incubated at 4°C in 1% FBS…..…….……….S4

**Fig. S4.** Quantitative fluorescence for peptides incubated with inhibitors…………………….S5

**Fig. S5.** Confocal imaging of W1 peptide with endocytosis inhibitors………………………..S6

**Fig. S6.** The frequency of fluorescence intensity for the W1 peptide……….…..……………..S7

**Fig. S7.** Quantitative fluorescence for peptides incubated with anti-GRP78…………………..S8

**Fig. S8.** Concentration-dependent siRNA transfection for GRP78 knockdown……………….S9

**Fig. S9.** RT qPCR data of GRP78 mRNA levels………………………………………………S10

**Fig. S10.** Western blot data of GRP78 protein levels………………………………………….S11

**Table S1**. List of sense and antisense primer sequences for qRT PCR………………………..S12


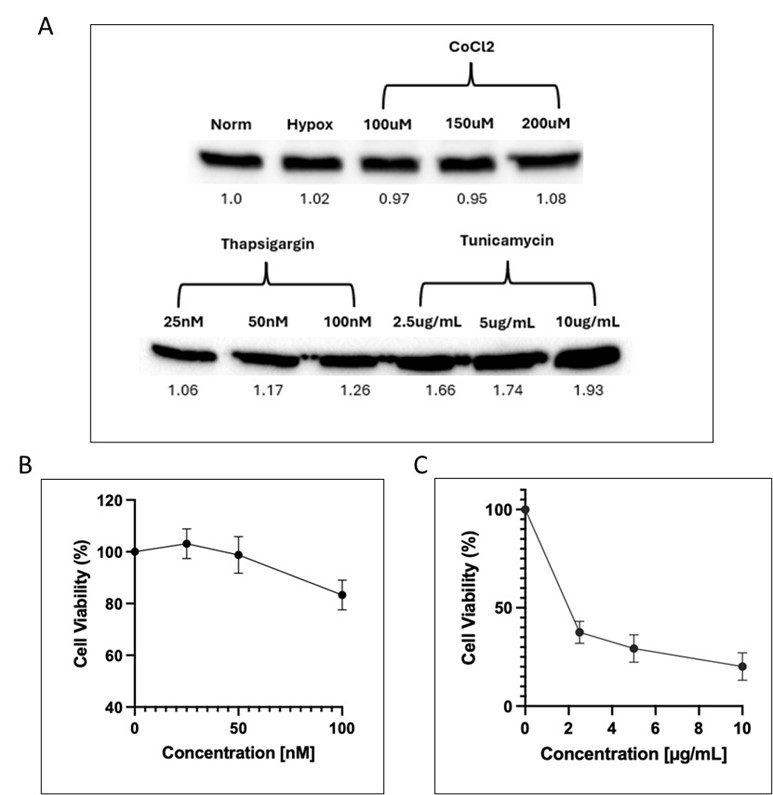


**Fig. S1.** (A) Western blot of the GRP78 expression levels within the DU145 cells under control (normoxia) and ER stress conditions. The cells were incubated under normoxia (21% O_2_) or hypoxia (5% O_2_), or increasing concentrations of CoCl_2_ [100, 150, & 200 µM], thapsigargin [25, 50, 100 nM], or tunicamycin [2.5, 5, 10 µg/mL] treatment for 24 hours in 10% FBS EMEM at 37^o^C. MTT cell viability assay for DU145 cells treated with (B) thapsigargin [25, 50, 100 nM] and (C) tunicamycin [2.5, 5, 10 µg/mL] for 24 hours in 10% FBS EMEM at 37˚C. Graphpad prism was used to generate the plots and analyze all statistical data.


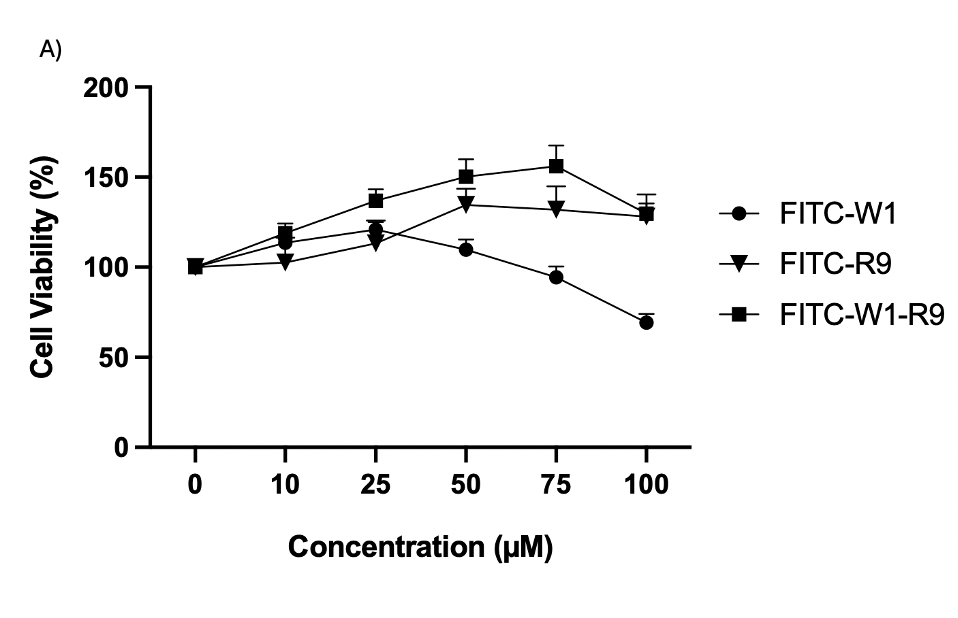

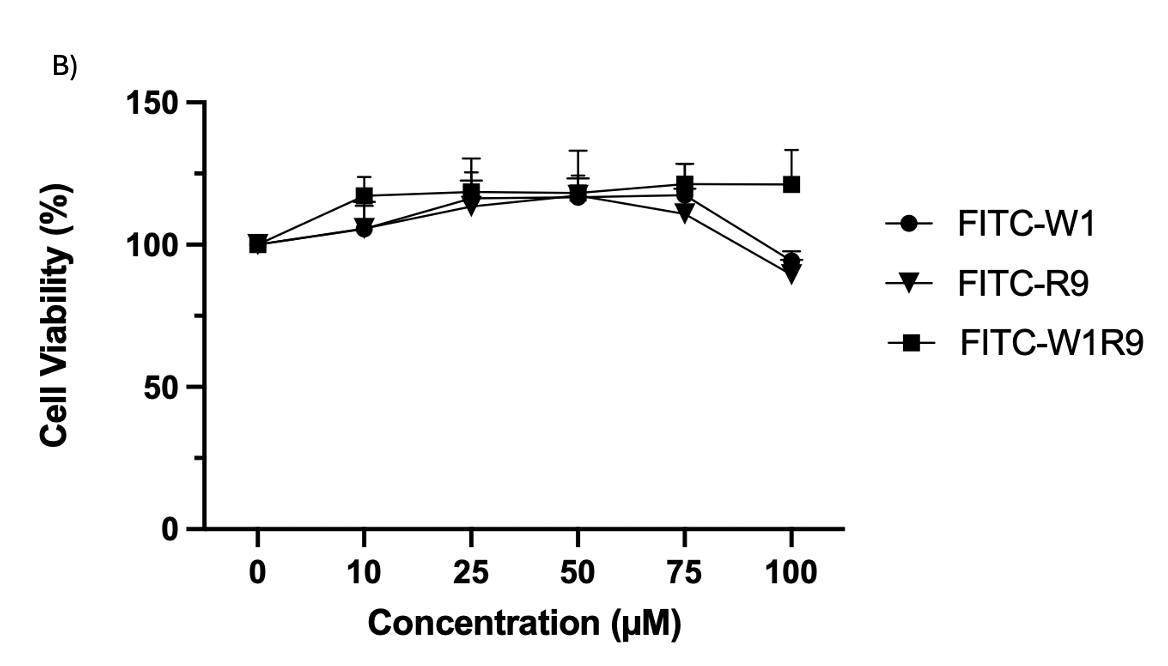


**Fig. S2.** MTT cell viability assay of peptides at various concentrations [0, 10, 25, 50, 75 and 100 µM] tested on the (A) DU145 prostate cancer cells and (B) WI-38 lung fibroblast cells for 24 hours of incubation time in 10% FBS in EMEM at 37°C. The error bars shown reflect the standard error of the mean. Graphpad prism was used to generate the plots and analyze all statistical data.


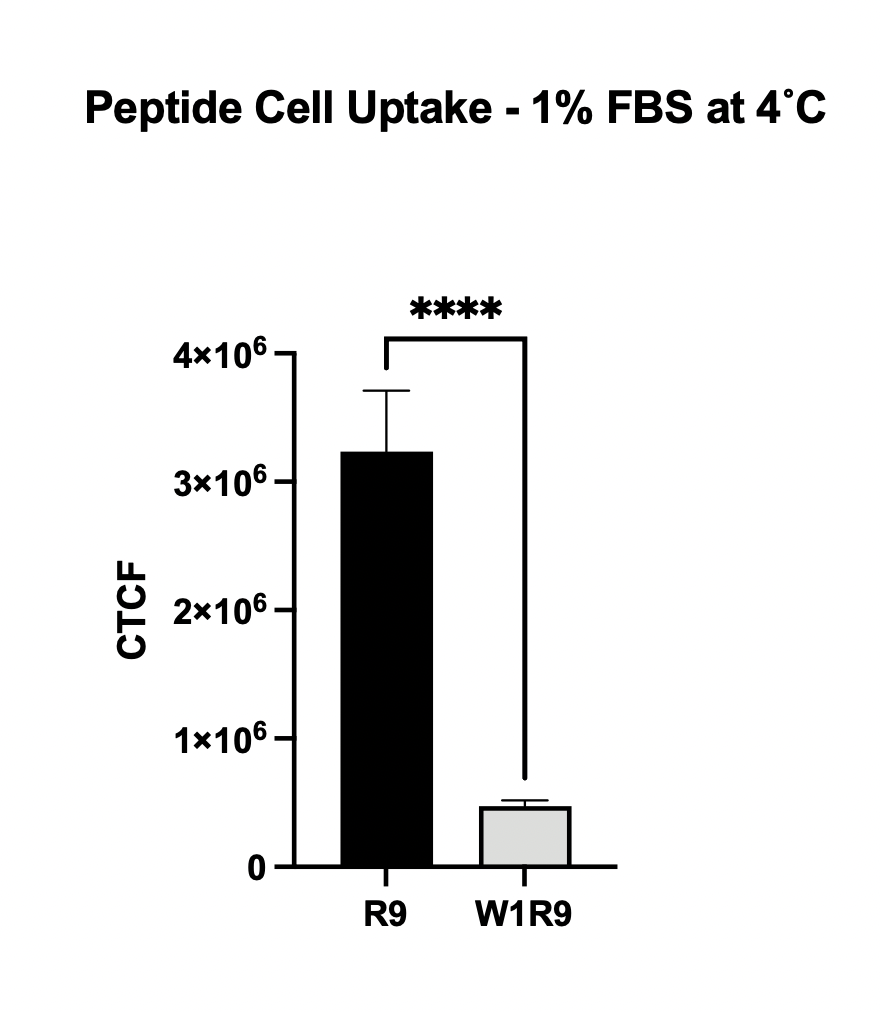


**Fig. S3.**  The corrected total cell fluorescence (CTCF) as a quantitative measurement of the fluorescence intensity of the FITC-labeled peptides (FITC-R9 and FITC-W1-R9) incubated at 4°C in 1% FBS with the DU145 cells. Data extrapolated from the confocal imaging (Figure 5) using Zen blue software. Statistical analysis and significance (two-tailed t-test P<0.0001) was accomplished by analyzing cells within each treatment using Fiji (ImageJ) and averaged, where the error bars reflect the standard error of the mean, showing significant decrease in fluorescence intensity for W1R9 relative to R9 peptide. Graphpad prism was used to generate the graph and analyze all statistical data.


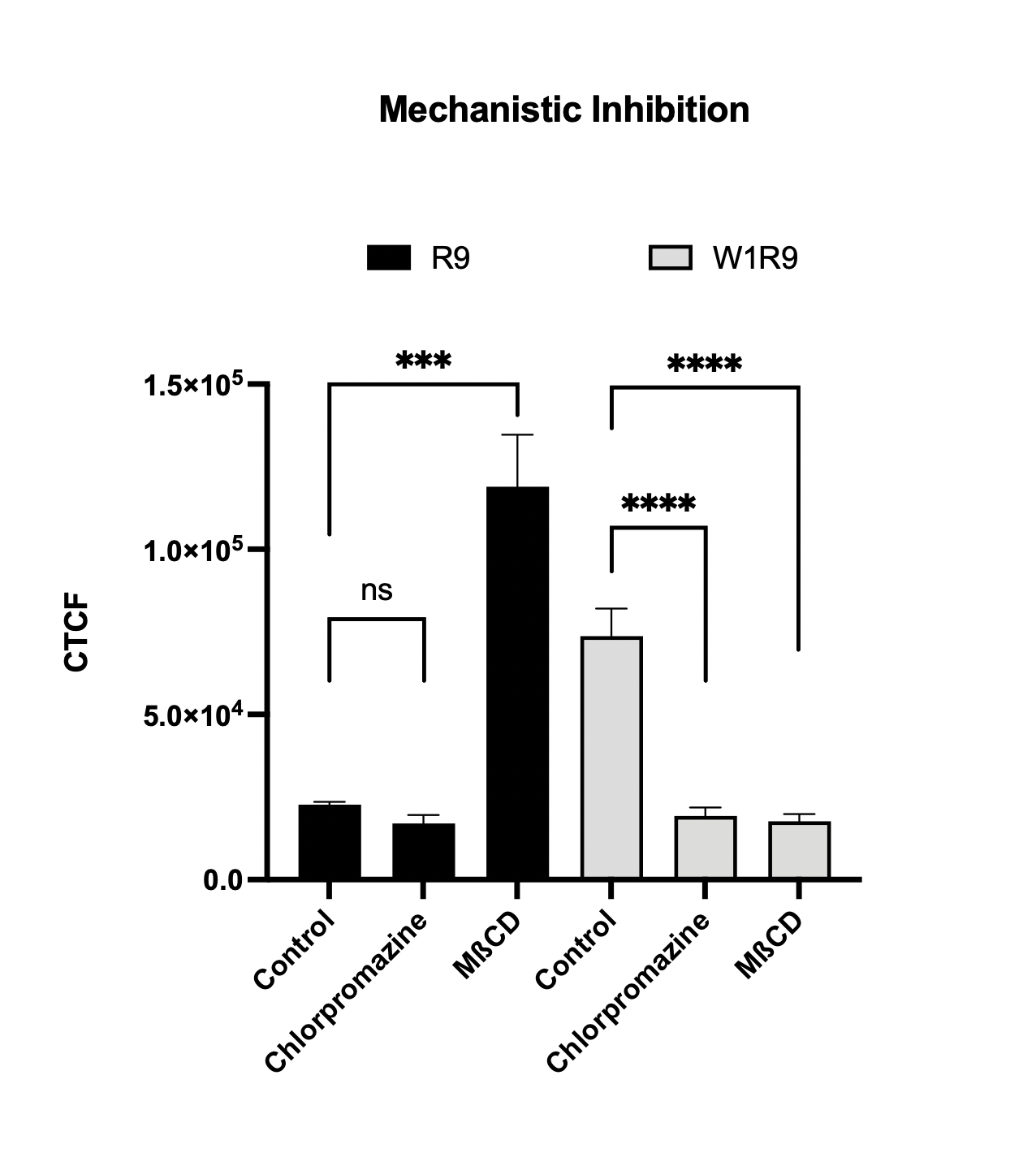


**Fig. S4.**  The corrected total cell fluorescence (CTCF) as a quantitative measurement of the fluorescence intensity of the FITC-labeled peptides (FITC-R9 and FITC-W1-R9) with the DU145 cells incubated with chemical inhibitors (chlorpromazine and methyl-ß-cyclodextrin) of receptor-meditated endocytosis. Data extrapolated from the confocal imaging (Figure 6) using Zen blue software. Statistical analysis and significance (two-tailed t-test P<0.001 and 0.0001 respectively) was accomplished by analyzing cells within each treatment using Fiji (ImageJ) and averaged, where the error bars reflect the standard error of the mean. Graphpad prism was used to generate the graph and analyze all statistical data.


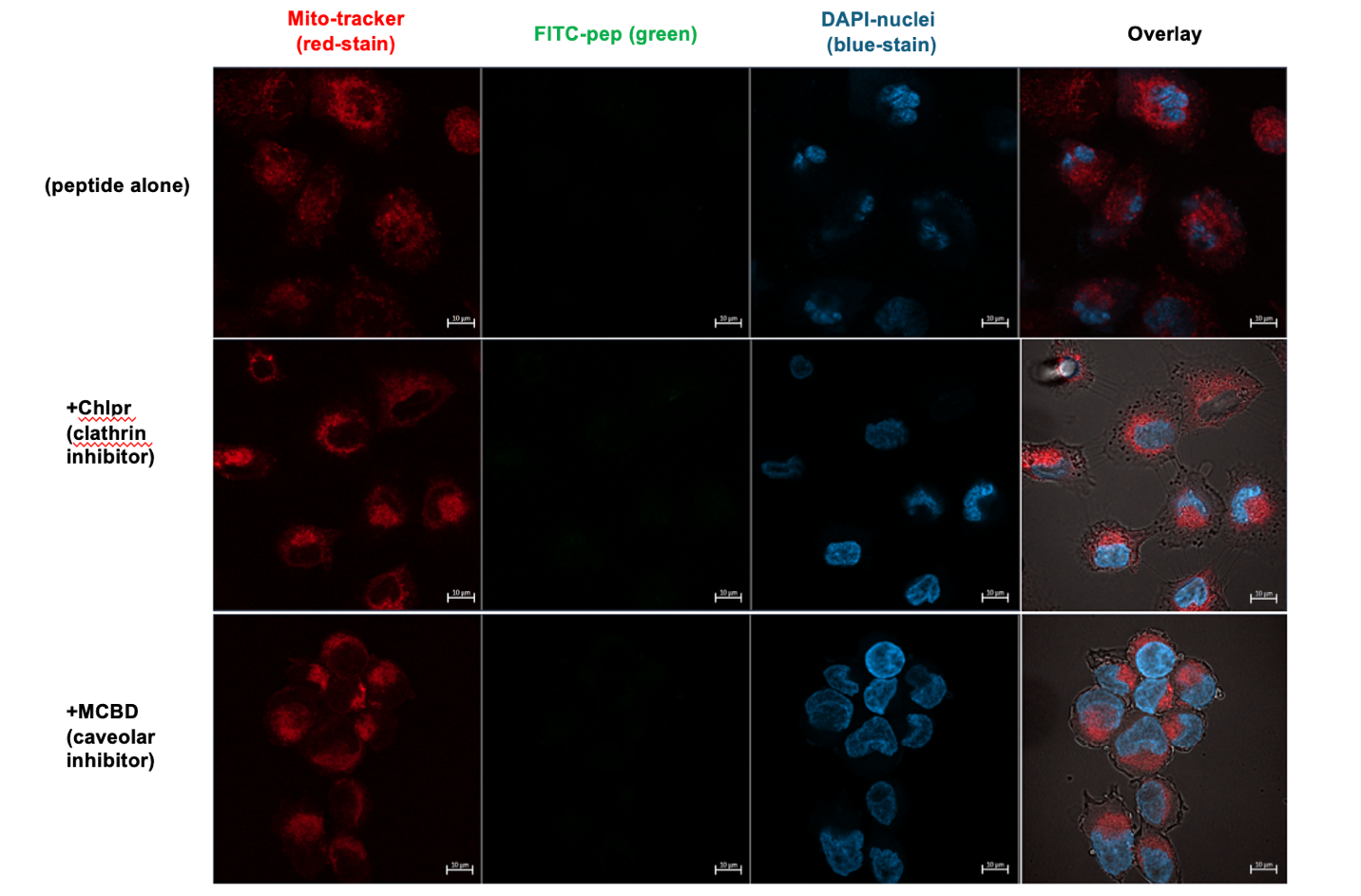


**Fig. S5.** DU145 cells treated with (10 µM) W1 peptide at 37˚C in FBS free EMEM for 30 minutes, in addition to various receptor mediated endocytosis inhibitors to determine peptide uptake mechanism. (A) is treated with W1 peptide alone as a baseline control, (B) treated with [5 mM] Methyl-ß-Cyclodextrin for 40 minutes, (C) treated with (30 µM) Chlorpromazine for 40 minutes.


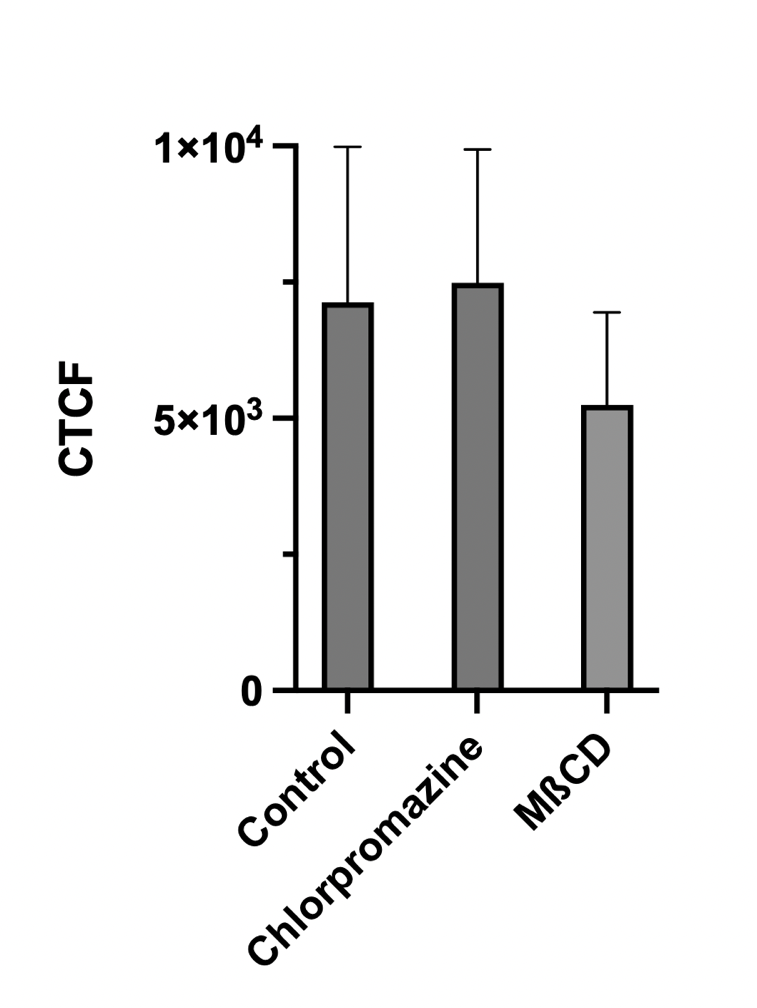


**Fig. S6.**  The corrected total cell fluorescence (CTCF) as a quantitative measurement of the fluorescence intensity of the targeting FITC-W1 peptide in the DU145 cells. Data extrapolated from the confocal imaging (Fig. S5) using Zen blue software. Statistical analysis showed no significance amongst the different treatments. Cell analysis within each treatment was done using Fiji (ImageJ) and averaged, where the error bars reflect the standard error of the mean. Graphpad prism was used to generate the graph and analyze all statistical data.


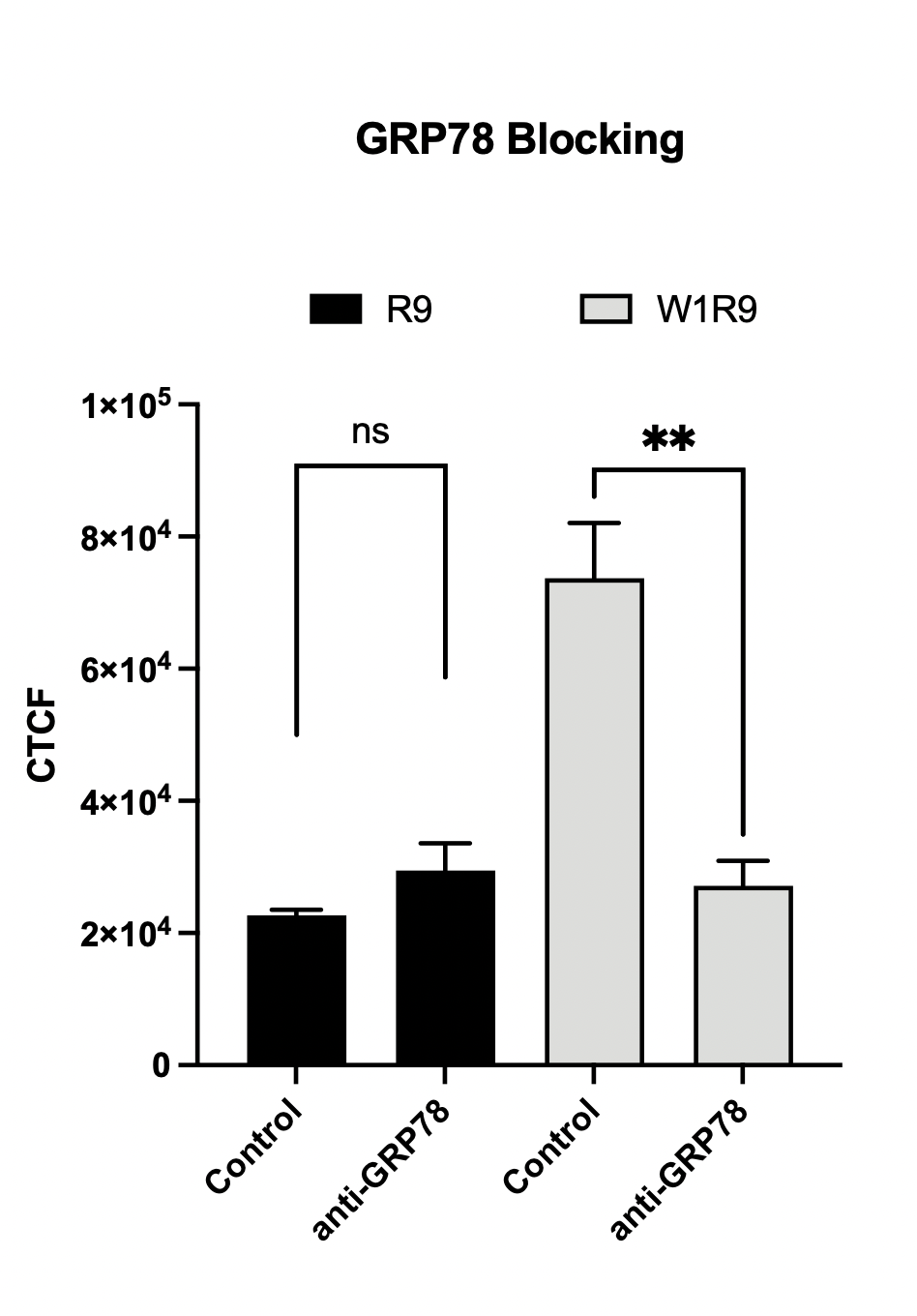


**Fig. S7.** The corrected total cell fluorescence (CTCF) as a quantitative measurement of the fluorescence intensity of the FITC-labeled peptides (FITC-R9 and FITC-W1-R9) with the DU145 cells incubated with anti-GRP78. Data extrapolated from the confocal imaging (Figure 7) using Zen blue software. Statistical analysis and significance (two-tailed t-test P<0.01) was accomplished by analyzing different cells within each treatment using Fiji (ImageJ) and averaged, where the error bars reflect the standard error of the mean. Graphpad prism was used to generate the graph and analyze all statistical data.


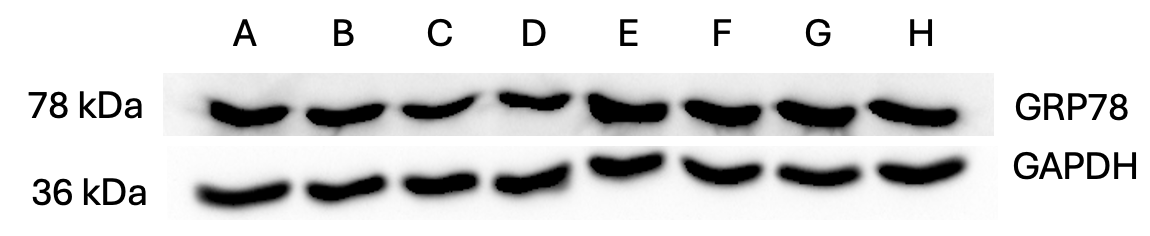


**Fig. S8.** Transfection study in the DU145 cells showing GRP78 protein downregulation with various siRNA concentrations in combination with RNAiMAX as the industry standard control, in comparison with cell penetrating peptide (R9) and the cell targeting and penetrating peptide W1-R9. Western blot of GRP78 normalized against GAPDH as loading control values. Transfection conditions include: (A) control, no treatment (B) RNAiMAX + siRNA [15 nM], (C) RNAiMAX + siRNA [25 nM], (D) RNAiMAX + siRNA [50 nM], (E) R9 + siRNA [25 nM], (F) R9 + siRNA [50 nM], (G) W1R9 + siRNA [25 nM], (H) R9 + siRNA [50 nM]. Peptides:siRNA were combined in a 5:1 N:P molar ratio.


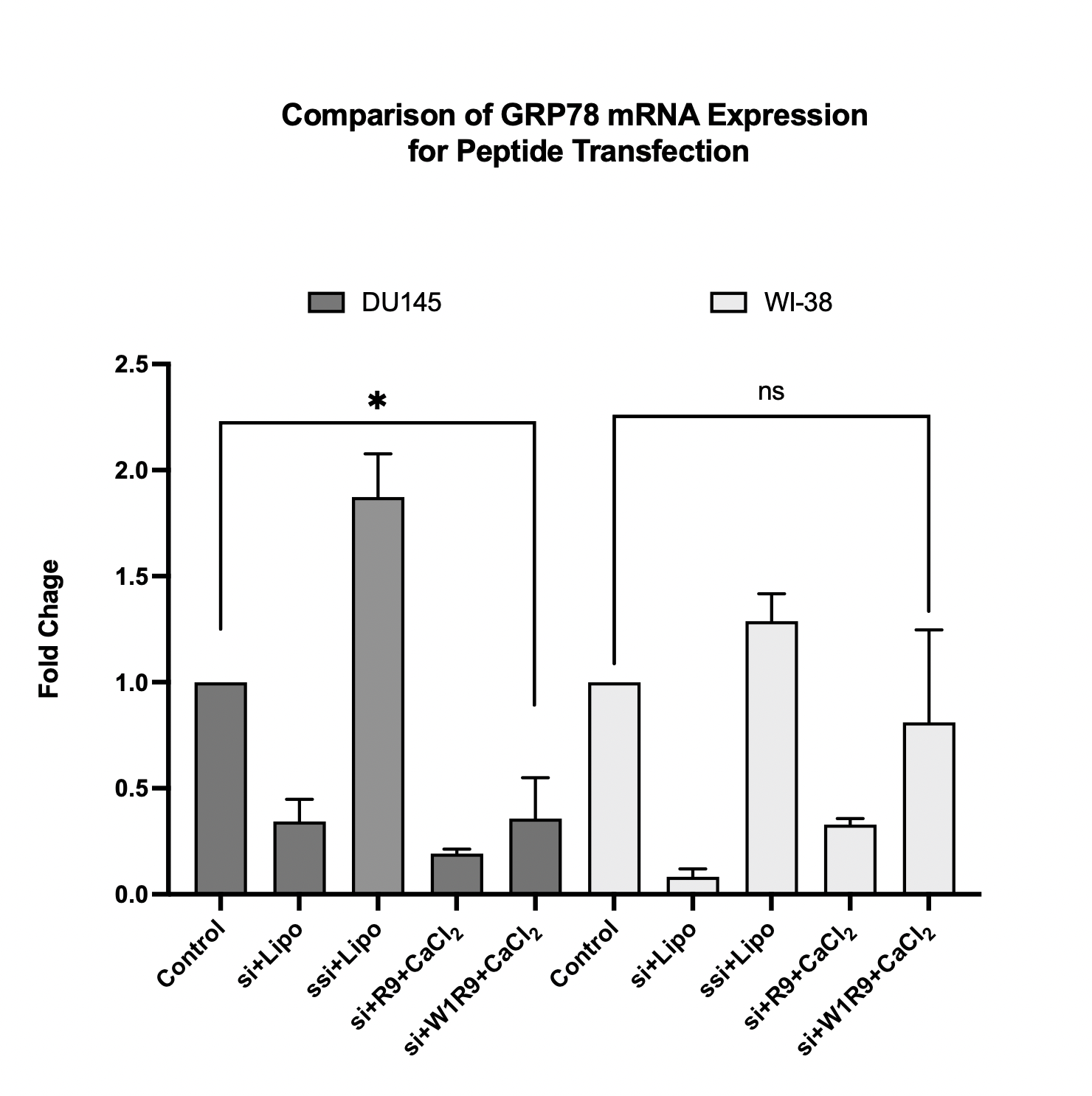


**Fig. S9.** Quantitative qRT PCR data relating the downregulation of GRP78 expression at the mRNA transcript levels. The peptides were incubated at [5:1 N:P] mole ratio relative to the siRNA. For transfections, the DU145 and WI-38 cells were incubated for 48 hours showing (from left to right): Control (no treatment), siRNA [25nM] + RNAiMAX Lipofectamine, scrambled siRNA (ssi, [25nM]) + RNAiMAX Lipofectamin, siRNA + R9 + CaCl_2_ [20 mM], and siRNA + W1R9 + CaCl_2_ [20 mM]. Statistical analysis and significance (two-tailed t-test P<0.05) was accomplished by analyzing different cells within each treatment using Fiji (ImageJ) and averaged, where the error bars reflect the standard error of the mean. Graphpad prism was used to generate the graph and analyze all statistical data.


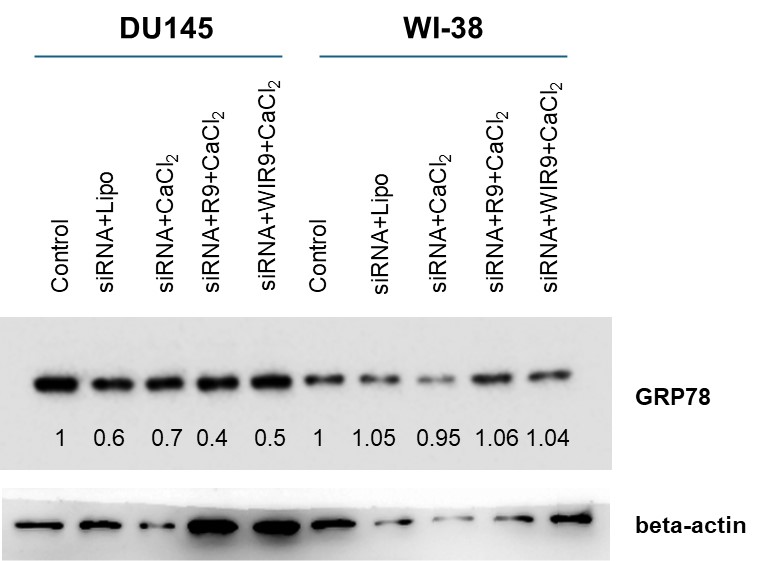


**Fig. S10.** Western blot relating the downregulation of GRP78 expression at the protein levels. The peptides were incubated at [5:1 N:P] mole ratio relative to the siRNA. For transfections, the DU145 or WI-38 cells were incubated for 48 hours with: (A) control, no treatment (B) RNAiMAX Lipofectamine (15µL) + siRNA [25nM], (C) siRNA+CaCl_2_ [20 mM], (D) siRNA+R9+CaCl2 and (E) siRNA + W1R9 + CaCl_2_.

**Table S1**. List of sense and antisense primer sequences for qRT PCR from Integrated DNA Technologies (IDT). The NM shows the reference sequence number taken from NCBI.

| **Primers** | **Sense Sequence** | **Antisense Sequence** |
| --- | --- | --- |
| GAPDH | 5’GGTGTGAACCATGAGAAGTATGA’3 | 5’GAGTCCTTCCACGATACCAAA’3 |
| GRP78 | 5’CCTTCGATGTGTCTCTTCTCAC’3 | 5’ACGCTGGTCAAAGTCTTCTC’3 |
| CHOP NM_001195053.1 | 5’CAAGAGGTCCTGTCTTCAGATG’3 | 5’GGGTCAAGAGTGGTGAAGATT’3 |
| Spliced XBP1 NM_001079539.2 | 5’GAGACAGAGAGCCAAGCTAATG’3 | 5’CAGGTTCTTCCTTCACTGAGAC’3 |
